# Venom gland transcriptome analyses of two freshwater stingrays (Myliobatiformes: Potamotrygonidae) from Brazil

**DOI:** 10.1038/srep21935

**Published:** 2016-02-26

**Authors:** Nelson Gomes de Oliveira Júnior, Gabriel da Rocha Fernandes, Marlon Henrique Cardoso, Fabrício F. Costa, Elizabete de Souza Cândido, Domingos Garrone Neto, Márcia Renata Mortari, Elisabeth Ferroni Schwartz, Octávio Luiz Franco, Sérgio Amorim de Alencar

**Affiliations:** 1 Programa de Pós-Graduação em Ciências Genômicas e Biotecnologia, Universidade Católica de Brasília, Brasília-DF, Brazil; 2 Centro de Análises Proteômicas e Bioquímicas, Pós-Graduação em Ciências Genômicas e Biotecnologia, Universidade Católica de Brasília, Brasília-DF, Brazil; 3 Departamento de Ciências Fisiológicas, Programa de Pós-Graduação em Biologia Animal, Universidade de Brasília, Brazil; 4 Programa de Pós-Graduação em Patologia Molecular, Universidade de Brasília, Brasília-DF, Brazil; 5 S-Inova Biotech, Pós-graduação em Biotecnologia, Universidade Católica Dom Bosco, Reitoria, Campo Grande, MS – Brazil; 6 UNESP – Universidade Estadual Paulista Júlio de Mesquita Filho, Campus Experimental de Registro, Registro, SP – Brazil

## Abstract

Stingrays commonly cause human envenoming related accidents in populations of the sea, near rivers and lakes. Transcriptomic profiles have been used to elucidate components of animal venom, since they are capable of providing molecular information on the biology of the animal and could have biomedical applications. In this study, we elucidated the transcriptomic profile of the venom glands from two different freshwater stingray species that are endemic to the Paraná-Paraguay basin in Brazil, *Potamotrygon amandae* and *Potamotrygon falkneri*. Using RNA-Seq, we identified species-specific transcripts and overlapping proteins in the venom gland of both species. Among the transcripts related with envenoming, high abundance of hyaluronidases was observed in both species. In addition, we built three-dimensional homology models based on several venom transcripts identified. Our study represents a significant improvement in the information about the venoms employed by these two species and their molecular characteristics. Moreover, the information generated by our group helps in a better understanding of the biology of freshwater cartilaginous fishes and offers clues for the development of clinical treatments for stingray envenoming in Brazil and around the world. Finally, our results might have biomedical implications in developing treatments for complex diseases.

The Potamotrygonidae family comprises the only group of Elasmobranchii restricted to freshwater environments, with their occurrence limited to some river systems of South America. This group is represented by four genera, among which *Potamotrygon* comprises the largest number of species with broad geographic distribution^1^.

The *Potamotrygon* genus includes benthic freshwater stingrays that are known for their long tail appendage with the presence of one to four serrated bone stings covered by a glandular epithelium whose cells produce venom^2^,^3^ (Fig. 1). Injuries caused by stingrays have always been present in riverine communities of inland waters and in South American coasts. Indeed, envenomation by stingrays is quite common in freshwater and marine fishing communities. Although having high morbidity, such injuries are neglected because they have low lethality and usually occur in remote areas, which favor the use of folk remedies^2^. Moreover, accidents are caused due to a reflex contact between stingrays and humans, yielding an animal tail whiplash and further leading to a sting introduction at the limb during direct contact, causing epithelial lining destruction and subsequent venom release^2^,^4^. Clinical manifestations occur by triggering painful processes and injuries, even producing ulcers and necrosis of affected tissues^2^. Erythema, edema and bleeding of different degrees around the sting site appear in the first poisoning stage following a characteristic cyanosis around the sting with a central necrosis, tissue slackness and the formation of a pink ulcer^2^.

Although several venom-producing animals have been studied across the globe, covering a wide spectrum ranging from aquatic to terrestrial environments, only a few reports have been made on stingrays^2^,^4^,^5^. Although there is currently no work on Potamotrygonidae transcriptomics, some molecules have been isolated in previous works from *Potamotrygon orbignyi,* such as the vasoconstrictor peptide orpotrin^6^ and porflan^7^, a peptide that is able to change vascular and capillary permeability^7^. Moreover, a single hyaluronidase was isolated from *Potamotrygon amandae*, which facilitates the absorption and diffusion of venom in the victim’s tissues, increasing systemic poisoning^5^. Also, recently, the venom gland transcriptome of a marine stingray, *Neotrygon kuhlii*, was generated^8^.

Due to the almost complete lack of information about freshwater stingray venoms, a transcriptomic characterization analysis could be a good strategy in order to elucidate components of the stingray venom mixtures, since it provides large amounts of information in a relatively short time^9^. In fact, venom gland transcriptomic analysis has been developed to study the venoms of organisms^10^,^11^. However, the venoms of aquatic animals have been poorly studied.

In this study, the venom gland transcriptome of two different freshwater stingrays, *Potamotrygon amandae* and *Potamotrygon falkneri*, was studied. Both species are endemic to the Paraná-Paraguay basin, in Brazil, and their specific identity is well corroborated^1^,^12^. The metabolic pathways in which venom compounds are produced were then described, providing novel insights into the venom production metabolism in river stingrays, elucidating the relationship between envenomation and the possible molecules associated. Additionally, specific transcripts were selected to do a three-dimensional structural analysis. We believe that our study will shed some light on the transcriptome composition of the venom gland from these animals and it could also have biomedical applications. Furthermore, this study presents the first transcriptomic analysis in freshwater stingrays.

## Results

### Sequencing and *de novo* assembly of the *P. amandae* and *P. falkneri* transcriptomes

The Illumina MiSeq sequencing of the *P. amandae* and *P. falkneri* cDNA libraries generated ~14.5 and ~15.4 million paired-end reads, with average lengths of 231 and 220 bp, respectively. After adapter trimming and quality filtering, we obtained a total of ~13.1 and ~13.7 million high-quality reads, with 92.7 and 92.4% of all bases having Phred (Q) scores above 33, respectively, which indicates good sequencing quality (Table 1). Using the Trinity *de novo* assembler software^13^, the high-quality reads were assembled into 147,881 and 105,191 contigs, respectively (Table 1). A length histogram of the *P. amandae* and *P. falkneri* assembled contigs shows that for both samples the majority of sequences (68.5 and 63.1%, respectively) ranged from 200 to 599 bp in length, and a considerable fraction (18.6 and 23.3%, respectively) were longer than 1 kb (Supplementary Fig. S1).

The assembled contigs of *P. amandae* and *P. falkneri* were then subjected to RSEM^14^ contig abundance analysis, followed by removal of contigs showing low expression (<1 FPKM) and short length (<200 bp). The use of this threshold value of FPKM was based on a RNA-Seq study with Trinity aimed to determine the threshold above which most biologically relevant transcripts are expressed^15^. Contigs shorter than 200 bp were discarded as likely to be uninformative. Then, the FPKM filtered contigs of *P. amandae* and *P. falkneri* were subjected to *in silico* candidate coding region identification using the TransDecoder software^15^, resulting in 25,092 and 22,083 contigs, with average lengths of 2,032 and 1,924 bp, respectively (Table 1).

### Transcriptome annotation

**Figure 2:**
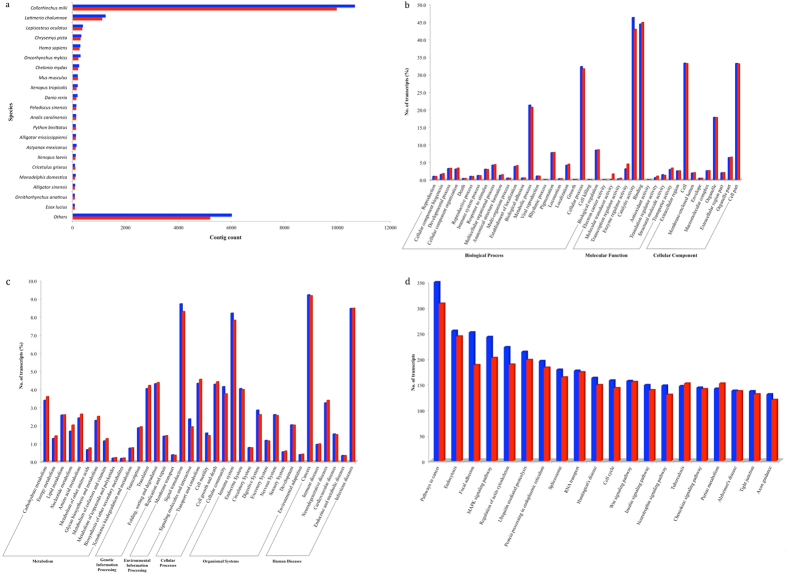
(**a**) Top-hit species distribution after BLASTx similarity search against the NCBI non-redundant (nr) database. (**b**) Gene Ontology classification of the transcripts separated in three categories: Biological Process, Molecular Function and Cellular Component (**c**). KEGG Classification of the transcripts separated in six categories: Metabolism, Genetic Information Processing, Environmental Information Processing, Cellular Process, Organismal Systems and Human Diseases. (**d**) Top 20 most abundant KEGG pathways are shown. *Potamotrygon amandae* and *P. falkneri* transcripts are represented in blue and red, respectively.

The *P. amandae* and *P. falkneri* filtered contigs were then analyzed for similarities with known sequences against the NCBI non-redundant (nr) peptide database^16^ using BLASTx^17^. This resulted in 21,245 (84.7%) and 19,316 (87.5%) significant hits annotated as similar to known proteins or matching known conserved hypothetical proteins, respectively (Table 2). Of these, 81.3 and 83.1% of the BLASTx hits have e-values of at least 1e^−40^ with the existing proteins at the NCBI nr database, and hits with e-value = 0 correspond to 31.8 and 31.6% of the total number of contigs, respectively (Supplementary Fig. S2). Based on the BLASTx hits for the *P. amandae* and *P. falkneri* assembled transcripts, the organism names for the top hits were extracted (Fig. 2a), which included matches to the following top four organisms: *Callorhinchus milii* (50.2 and 51.6%), *Latimeria chalumnae* (5.9 and 5.8%), *Lepisosteus oculatus* (1.8 and 1.9%) and *Chrysemys picta* (1.5 and 1.6%), respectively. In the top 21 organisms shown in Fig. 2a, only seven are fish species that have known genome sequences. This explains the BLASTx hits to more distant species, which is similar to data found in other transcriptomic assemblies of fish species^18^,^19^.

We further annotated the *P. amandae* and *P. falkneri* assembled transcripts with BLASTx against the UniProtKB database. A total of 17,250 (68.8%) and 15,935 (72.2%) transcripts matched to already reported sequences in UniProtKB, respectively (Table 2). Considering the NCBI and UniProtKB BLASTx searches, a total of 3,387 (15.3%) *P. amandae* and 2,767 (12.5%) *P. falkneri* transcripts did not result in hits against any of these databases. A closer analysis of these transcripts showed that 3,773 *P. amandae* and 2,735 *P. falkneri* harboured an ORF > = 100 amino acids, which suggests that a significant proportion of these could correspond to putative *P. amandae* and *P. falkneri*-specific transcripts.

In order to obtain more details about the classification of the proteins, the UniProtKB hits obtained from the BLASTx analysis for both species was crossed against the InterPro database. A total of 16,961 (67.6%) *P. amandae* and 15,666 (70.9%) *P. falkneri* assembled transcripts had hits with InterPro entries (Table 2). For *P. amandae*, the most abundant hits were P-loop containing nucleoside triphosphate hydrolase (1168 counts), protein kinase-like domain (746 counts) and zinc finger, C2H2-like (710 counts) (Supplementary Table S1). Similarly, the most abundant hit for *P. falkneri* was also P-loop containing nucleoside triphosphate hydrolase (1076 counts), followed by zinc finger, RING/FYVE/PHD-type (679 counts) and protein kinase-like domain (674 counts) (Supplementary Table S2).

The *P. amandae* and *P. falkneri* assembled transcripts which did not result in coding region identification using the TransDecoder software were further analyzed by the PLEK software for the prediction of long non-coding RNAs (lncRNAs)^20^. This class of transcripts is involved in important biological processes, such as dosage compensation, regulation of gene expression and cell cycle regulation^21^. This analysis resulted in the prediction of 1,913 *P. amandae* and 1,611 *P. falkneri* putative lncRNAs (Table 2).

### Functional and pathway annotations

Gene Ontology (GO) analysis was performed in order to classify the functions of the annotated transcripts. A total of 25,092 *P. amandae* and 22,083 *P. falkneri* assembled transcripts were mapped into three categories: biological process, molecular function and cellular components. This analysis resulted in at least one GO term assigned to 64.9 and 69.7% of the transcripts, respectively (Table 2).

*Potamotrygon amandae* and *P. falkneri* predicted proteins assigned to biological processes were mainly associated with cellular processes, metabolic processes, and biological regulation processes (Fig. 2b). The distribution of the GO terms assigned to molecular functions was reasonably similar to those obtained in the assembled transcriptome of *Neotrygon kuhlii*^8^, mainly linked to catalytic activity and binding. Finally, terms assigned to cellular components included cellular locations, cell part and organelles. GO terms for the three categories showed a homogenous distribution between *P. amandae* and *P. falkneri* transcripts, with the exception of catalytic activity, which was slightly higher in *P. amandae.*

The *P. amandae* and *P. falkneri* assembled transcriptomes were further annotated by mapping the UniProt accession numbers of the transcripts (obtained from running BLASTx against UniProtKB) onto pathways in KEGG^22^. A total of 14,131 *P. amandae* transcripts and 13,147 *P. falkneri* transcripts were assigned to 269 KEGG pathways corresponding to six categories: “Metabolism”, “Genetic Information Processing”, “Environmental Information Processing”, “Cellular Processes”, “Organismal Systems” and “Human Diseases”. The percentage of assigned transcripts for each of the 37 pathways in these categories is shown in Fig. 2c. It can be seen that the pattern of distribution of the functional categories is similar for the *P. amandae* and *P. falkneri* transcriptomes, and that the most representative clusters are “Cancers”, “Signal transduction”, “Immune system” and “Infectious diseases”. Interestingly, “Pathways in cancer” were the most abundantly assigned pathways for the *P. amandae* and *P. falkneri* transcriptomes, followed by “Endocystosis”, “Focal adhesion”, “MAPK signaling pathway”, “Regulation of actin cytoskeleton” and “Ubiquitin mediated proteolysis” (Fig. 2d).

The metabolism category involves all metabolic pathways present in the stingray spine, including those involved with amino acids, carbohydrate and lipid metabolism, which constitute 51% of all metabolism classes. Within the amino acid metabolism, there were six pathways associated with biosynthetic amino acid pathways in *P. amandae* and 10 in *P. falkneri.* Moreover, 113 transcripts from both species were associated to the same amino acid pathways, such as lysine degradation (Supplementary Fig. S3), metabolism of arginine and proline (Supplementary Fig. S4), cysteine and methionine metabolism (Supplementary Fig. S5), and glycine, serine and threonine metabolism (Supplementary Fig. S6). These metabolic pathways clearly show the importance of protein turnover, which could be directly involved in venom protein production.

In the metabolic analysis of carbohydrate pathways, 11 transcripts were exclusive to *P. amandae* and 14 to *P. falkneri*, and 175 transcripts were shared between both stingrays. Among them, pathways related to the citrate cycle (Supplementary Fig. S7), glycolysis (Supplementary Fig. S8) and pyruvate metabolism (Supplementary Fig. S9) were observed. Among lipid metabolism, 16 transcripts were restricted to *P. amandae* and 143 were shared between both species, including fatty acid degradation pathways (Supplementary Fig. S10) and metabolism of sphingolipids and glycerophospholipids (Supplementary Figs S11 and S12), being important in cellular degradation.

The SignalP 4.1 software^23^ was used in order to predict the presence of signal peptide cleavage sites within the assembled transcriptome peptide sequences of both stingrays. A total of 1,441 *P. amandae* and 1,185 *P. falkneri* peptide sequences were predicted to contain a signal peptide cleavage site by SignalP (Table 2).

### Comparative analysis among four fish species

**Figure 3:**
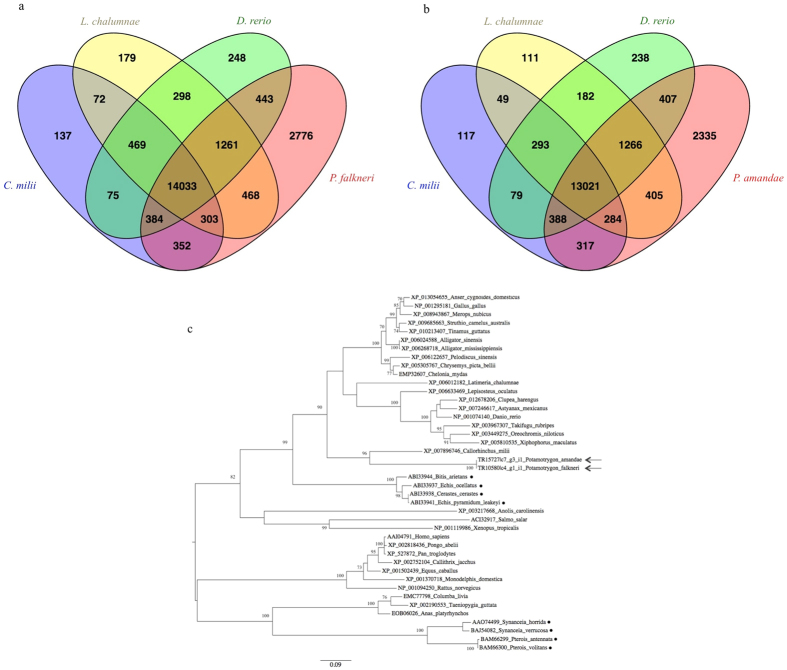
Venn diagram showing the comparison of the *Potamotrygon amandae* transcriptome (**a**) against *Callorhincus milii*, *Latimeria chalumnae*, *Danio rerio* and *P. falkneri* UniProtKB proteins and the comparison of the *P. falkneri* transcriptome (**b**) against *C. milii*, *L. chalumnae*, *D. rerio* and *P. amandae* UniProtKB proteins. (**c**) Phylogenetic tree of selected hyaluronidase homologues from various animal species. The tree was constructed by the Maximum Likelihood method in MEGA v.6 with 500 replicates, and involved 42 amino acid sequences (UniProtKB accession numbers are shown for each protein). The percentage of bootstrap confidence values is shown at the nodes (only values > 70% are shown). The tree is drawn to scale, with branch lengths measured in the number of substitutions per site. The proteins of *P. amandae* and *P. falkneri* are pointed by arrows, and black spots indicate the hyaluronidases from venomous organisms.

The UniProtKB database^24^ currently provides peptide sequences of several fish species which were obtained from transcriptomic studies. Therefore, using BLASTx we compared the assembled transcriptomes of *P. amandae* and *P. falkneri* against each other, and also against the peptide sequences of two cartilaginous fishes (*Callorhincus milii* and *Latimeria chalumnae*) and *Danio rerio*, the model fish (Fig. 3a,b). A total of 14,033 transcripts expressed in *P. amandae* were shared among *C. milii*, *L. chalumnae*, *D. rerio* and *P. falkneri* (Fig. 3a), and 13,021 *P. falkneri* transcripts were shared among *C. milii*, *L. chalumnae*, *D. rerio* and *P. amandae* (Fig. 3b). As expected, a significant proportion of transcripts (2,776 in *P. amandae* and 2,335 in *P. falkneri*) are shared only between the two stingray species. A search for transcripts that were unique in the stingray species resulted in 3,594 *P. amandae* unique transcripts and 2,592 *P. falkneri* unique transcripts. However, only a small proportion of these unique transcripts (208 in *P. amandae* and 182 in *P. falkneri*) had BLASTx hits against the UniProtKB database. Most of the unique *P. amandae* transcripts were mapped to “Translation”, “Transport and Catabolism”, “Infectious diseases” and “Carbohydrate Metabolism” KEGG functional categories, whereas the *P. falkneri* unique transcripts were mostly mapped to “Translation”, “Cancers”, “Amino Acid Metabolism” and “Folding, Sorting and Degradation” KEGG functional categories.

### *Potamotrygon amandae* and *P. falkneri* transcript hits to UniProtKB Tox-Prot database

In order to evaluate the transcripts related to animal toxin/venoms, a BLASTx analysis was made against the UniProtKB/Swiss-Prot Tox-Prot database^25^. Several proteins related to the toxins/venoms of many animals were found in this search, such as phospholipase A2, metalloproteinases, c-type lectin, hyaluronidase, serine-proteinases and L-amino acid oxidases, which were ranked in terms of expression abundance in *P. amandae* and *P. falkneri* (Tables 3 and 4, respectively).

The identifiable toxin transcripts for *P. amandae* and *P. falkneri* represent 111 and 115 PFAM families, respectively. The highest expressed family for both stingray species was glycoside hydrolase, which includes hyaluronidase. This transcript accounted for 63.8 and 44% of the total FPKM for *P. amandae* and *P. falkneri*, respectively. The toxin transcript abundances are followed by the CAP protein family (5.7%), EF-hand protein (4.3%), translationally controlled protein (3.5%) and the MANEC domain family in the *P. amandae* transcriptome, and EF-hand protein (5.6%), sea anemone cytotoxic protein family (3.8%), translationally controlled tumour protein (3.4%) and the MANEC domain family (2.9%) in the *P. falkneri* transcriptome.

In order to better characterize the *P. amandae* and *P. falkneri* highly expressed hyaluronidase transcript, a phylogenetic analysis was carried out to discern evolutionary relationships among representative hyaluronidases from a diverse set of organisms. Similarly to the hyaluronidase-like transcript previously identified in the venom gland of the marine stingray *N. kuhlii*^8^, the phylogenetic analysis of the *P. amandae* and *P. falkneri* hyaluronidase transcripts also revealed that they were non-monophyletic to hyaluronidases previously molecularly characterised from other venomous organisms, such as different fishes and snakes (Fig. 3c).

### Theoretical 3D Structural Analysis

**Figure 4:**
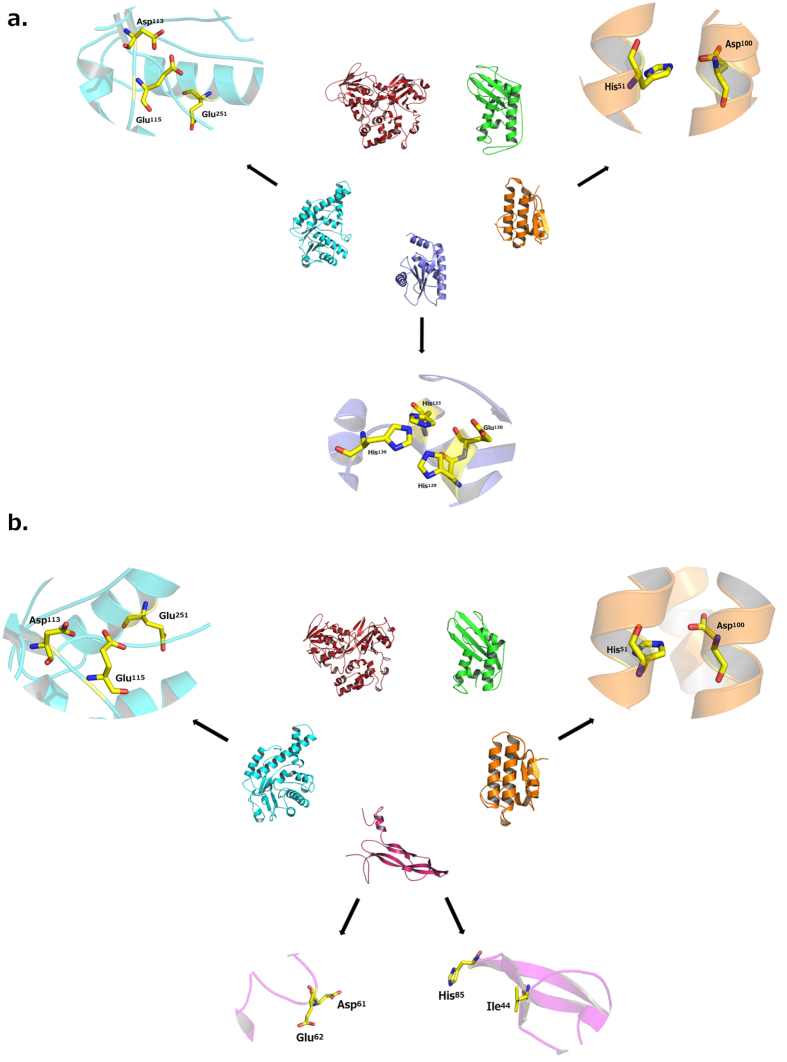
(**a**) Three-dimensional theoretical structures for cysteine-rich secretory protein (CRISP) (green), phospholipase A2 (orange), metalloproteinase (purple), hyaluronidase (cyan) and L-amino acid oxidase (red) from *Potamotrygon amandae*. The yellow sticks are highlighting the conserved amino acid residues that might display a crucial role in the mechanisms of action of their respective proteins. (**b**) Three-dimensional theoretical protein structures for cysteine-rich secretory protein (CRISP) (green), phospholipase A2 (orange), vascular endothelial growth factor A (VEGFA) (pink), hyaluronidase (cyan) and L-amino acid oxidase (red) from *P. falkneri*. The yellow sticks are highlighting the conserved amino acid residues that might display a crucial role in the mechanisms of action of their respective proteins.

In order to assess the three-dimensional theoretical structures of some protein families identified in the stingray transcriptome, as well as to observe the disposition of their catalytic domains, molecular modeling studies were carried out for several predicted toxin transcripts from both stingray species (Fig. 4). BLAST results showed that the selected toxin transcript sequences possessed reliable alignments with template sequences presenting high scores, identity and coverage, as well as low E-values and, for this reason, they were used for further analysis. In this context, a hundred three-dimensional homology models were built for the folowing families: phospholipase A2, serine protease, cysteine-rich secretory protein (CRISP), glycoside hydrolase (hyaluronidase), flavin-containing amine oxidoreductase (L-amino acid oxidase), C-type lectin (CLEC) and vascular endothelial growth factor A (VEGFA) for both *P. amandae* and *P. falkneri*, and disintegrin (snake venom metalloproteinase) only for *P. amandae*. When evaluated by PROCHECK, these 15 models presented G-factor values, which correspond to the average score for the dihedral angles added to the covalent forces obtained for the main chain, between −0.01 and −0.5. Moreover, Ramachandran’s plot revealed that all structures presented more than 80% of their amino acid residues in the most favorable regions. The ProSa server also confirmed that the Z-scores for these theoretical models are in agreement with Z-scores obtained for proteins structurally resolved by X-ray crystallography or nuclear magnetic resonance (NMR) techniques available in protein databases. As exceptions, for C-type lectins from both *P. amandae* and *P. falkneri*, and for VEGFA from *P. amandae*, it was observed that their theoretical structures were not in agreement with the parameters used for validation procedures. This might be due to an extensive random tail located at their C-terminal region, which did not adopt any stable conformation even after refinement and energy minimization procedures. Due to this, 12 theoretical models with good quality were generated for both stingray species. The physicochemical properties for these models, such as isoelectric point (IP), number of disulfide bonds, solvent-accessible surface area (SASA), as well as α-helix, β-sheet and loop contents, are all summarized in Supplementary Table S3. Among these models, it was also observed that hyaluronidase, CRISP and phospholipase A2 from both *P. amandae* and *P. falkneri*, snake venom metalloproteinase from *P. amandae* and VEGFA from *P. falkneri* presented conserved residues also reported in the literature, and that might play a crucial role in the mechanisms of action of their respective proteins.

Taking into account the great importance of some amino acid residues for the correct activity of such proteins, all the reliable theoretical models were superimposed on their template structures, as well as with other members from each enzyme class available in databases, in order to identify conserved residues between them, also highlighting the influence of these residues in the physicochemical properties of the active sites. As observed in Fig. 4, the predicted hyaluronidases, CRISP and phospholipases A2 from both stingray species, as well as VEGFA from *P. falkneri* and snake venom metalloproteinase from *P. amandae*, had their activity-related and conserved amino acid residues highlighted, which have been previously described as being crucial elements for the function of these proteins. On the other hand, no residue-related activity was found for L-amino acid oxidase from either stingray species when compared with their template structures or with other similar proteins from databases.

## Discussion

The study of stingray transcriptomics is challenging, since there has been little information about this organism published in the literature over the last few years, with freshwater species of the family Potamotrygonidae being completely unexplored. Here, two stingray species were compared according to their transcriptome profiles, showing enormous similarities between them. The single transcriptomic analysis from cartilaginous fish described until now was carried out with the marine blue-spotted stingray, *Neotrygon kuhlii*^8^. Notably, the total number of contig sequences obtained for *P. amandae* and *P. falkneri* in our work is elevated when compared to the previously assembled *N. kuhlii* transcriptome, which resulted in only 4,584 contigs^8^. This is probably due to the higher sequencing coverage done in the present study. Also, our sequence data provides a large number of transcripts for the *Potamotrygon* genus when compared to publicly available data from the Genbank database where only 255 nucleotide sequences are listed for this genus, mostly from mitochondria.

Furthermore, in the transcriptomic analysis of *N. kuhliii*, no phospholipase, metalloproteinase or hyaluronidase transcripts were identified. In the freshwater stingrays’ transcriptomic study, the number of transcripts related to venom toxins is much more significant than in *N. kuhliii*, which might be due to a low sequencing coverage in the *N. kuhliii* transcriptomics study, or perhaps because freshwater stingray species are in fact richer in venom toxins than marine stingrays.

According to the KEGG analysis from this study, an interesting issue is that metabolic pathways represent a large part of the classes of transcripts found for both stingray species. As mentioned above, the three main pathways associated with metabolism were those related to the metabolism of amino acids, carbohydrates and lipids (Fig. 2c). The main aspect that may be addressed when it comes to amino acid metabolism is protein production. Improvement in protein production leads to venom production, which is used in defense and predation by venomous animals^26^.

Moreover, polysaccharides were also observed due to their importance in the production of venom by glands, although glycosylation is widely considered a common modification in numerous venom proteins and impacts on their *in vivo* venomic functions^27^. Snake venoms, for example, have a large amount of glycoproteins with carbohydrates attached to the N-terminal, and these glycosylations are extremely important as they ensure correct folding of the functional domains^27^.

Several protein families related to envenomation were also observed. The prothrombim activator factors account for the procoagulant nature of the venoms that cause disseminated intravascular coagulopathy (DIC), by activating zymogens in the coagulation cascade^28^. These proteinases activate the cascade at specific steps because they are analogous to endogenous coagulation factors. Based on their specificity, they could be characterized as factor X activators, prothrombin activators and thrombin-like enzymes^29^. These enzymes, as well as the others mentioned, represent the great main causes of envenomation with stingrays, since thrombi formed by them may result in more advanced symptoms.

The most highly expressed transcript in both stingray species was hyaluronidase. At the venom composition level, the hyaluronidases are extremely important since they can improve the diffusion of fluids across the skin by hyaluronan degradation, contributing to venom dissemination, potentiating systemic envenomation and demonstrating great potential in local hemorrhage. Similar effects were observed for the *Naja naja* venom^30^. Among all toxin transcript families described here, the glycoside hydrolase family, which includes hyaluronidase, is one of the few already previously described in the literature on stingrays^5^. The symptoms described in *P. amandae* and *P. falkneri* envenomations are also conspicuous in hyaluronidase activity^5^.

Phospholipases A2 play a pivotal role in the biosynthesis of prostaglandin and other inflammation mediators, and are abundant in many animal venoms, holding both toxic and digestive characters. They have a vast spectrum of activities, such as neurotoxic, myotoxic, hemolytic, edematogenic, hyperalgesic, pro-inflammatory, hypotensive, platelet-aggregation inhibition, anticoagulant and cytotoxic, being one the most dangerous toxins in animals^31^. These enzymes have already been described in stingray venom from *Dasyatis pastinaca*^32^. Like metalloproteinases, these enzymes are very dangerous in stingray envenoming related accidents and follow the symptomatology that is characterized by hemorrhage and necrosis in the victim’s affected member^32^.

The proteinases represent the most dangerous venom classes in all venomous organisms because of their characteristic symptoms, which involve severe pain, necrosis and, in some cases, amputation of the affected limbs. Metalloproteinases are normally zinc-dependent enzymes, which play many roles in several venomous animals, such as hemorrhage induction^33^, inhibition of platelet aggregation^33^, myonecrosis^34^, and inflammatory responses^34^. In addition to hemorrhagic activity, members of the snake venom metalloproteins (SVMP) family also have fibrinogenolytic activity, act as prothrombin activators, activate blood coagulation factor X, possess apoptotic activity, inhibit platelet aggregation, and are pro-inflammatory and inactivate blood serine proteinase inhibitors^35^.

The serine protease and venom prothrombin activator have the same complexity as the cysteine-rich secretory protein/venom allergen factor. Both of these classes have a characteristic trypsin-like serine protease domain^36^. These serine proteases may act on coagulation cascade components, fibrinolytic and kallikrein–kinin systems, and they may cause imbalance of the hemostatic system in cells^36^. The venom growth nerve factor (VGNF) transcript was also identified for both stingrays. Several VGNF activities have been investigated, including rapid change in phospholipid metabolism, change in ion flux across the plasma membrane and phosphorylation of specific proteins, as well as synthesis induction and release of neurotransmitters, symptoms observed for the pit viper *Crotalus adamanteus* venom^37^. This factor also induced weak vascular permeability, suggesting its involvement in the hemorrhagic activity of the venom of *Viperaxantina paslestinae*^38^.

Other venom families described here were the cysteine-rich secretory proteins (CRISPs). The CRISPs are glycoproteins found exclusively in vertebrates and have diverse functions^39^. In mammalian reproduction and reptilian venom, they are hypothesized to disrupt the homeostasis of their prey across several mechanisms, including blockage of cyclic nucleotide-gated and voltage-gated ion channels and inhibition of smooth muscle contraction^39^.

Two neurotoxins were found in both stingrays’ transcriptomes: ohanin and α-atrotoxin-Lt1a. Neurotoxins may act in neuromuscular transmission where the neurotransmitter is acetylcholine, but on some occasions these toxins may interfere with neurotransmitters such as adrenaline, dopamine, GABA, noradrenaline, and γ-aminobutyrate^40^. Ohanin has been purified from *Ophiophagus hannah* (king cobra) venom, and it was shown to induce hypolocomotion and hyperalgesia in mice, suggesting its action through the central nervous system^40^. α-Latrotoxin-Lt1a is a neurotoxin from the European black widow spider (*Latrodectus tredecimguttatus*), which causes massive vesicle exocytosis leading to muscle fasciculation, tremor and muscle paralysis^41^.

In addition to gathering information about the biology of these organisms, studies generating transcriptome analyses of venom glands have several biomedical applications. For example, understanding the variations in protein components is instrumental in interpreting clinical symptoms during human envenomation and in searching for novel venom proteins with potential therapeutic applications^42^. In the last decade, transcriptomic analyses of venom glands have helped in understanding the composition of various snake venoms in great detail^42^. Additionally, proteins from venoms of different organisms have been used to develop drugs specific for complex diseases such as cancer^43^. In our study, it is noteworthy the fact that we identified in our KEGG analysis (Fig. 2c,d) several transcripts that are classified as “disease related”, especially for cancer, neurodegenerative and infectious diseases. In that regard, it is well recognized that animal venoms have been potentially rich sources of biologically active biomolecules that could be mined for the discovery of drugs, drug leads and/or biosimilars^44^. A proof of concept is a novel approach to explore venoms for potential biosimilarity to other drugs based on their ability to alter the transcriptomes of test cell lines followed by informatic searches and using the Connectivity Mapping to match the action of the venom on the cell gene expression to that of other drugs in the Connectivity Map database^44^. Thus, we believe that the transcripts identified in our study could be further evaluated for biomedical applications, especially those related to drug development and new therapies for complex diseases.

### Conclusions and Future Prospects

In summary, the transcriptomes of two freshwater stingray venoms were described, and several transcripts were presented, and their possible relationship with the envenoming and venom of different animals was discussed here. Different types of putative toxins correlate to the symptomatology present in riverine population affected by these accidents. For this reason, the knowledge and study of these species are extremely important, since these populations are completely neglected and literature has just little description on the molecular mechanisms of these accidents. On the other hand, this study also represents significant gains (or improvements) in the information about the *Potamotrygon* genus, which was described for the first time using transcriptomic analytics’ tools. Our study also helps in a better understanding on the biology of freshwater cartilaginous fishes and could have several biomedical applications for drug development studies.

## Materials and Methods

### Stingray spine collection and RNA isolation

Spine samples from two species of freshwater stingrays, *Potamotrygon amandae* (n = 3 specimens) and *Potamotrygon falkneri* (n = 3 specimens), were collected from the Paraná River, in the state of Mato Grosso do Sul, Midwest of Brazil (about 20°47′S 51°37′W). Since the spines regenerate, all specimens captured were released after extraction of the spine. All collection procedures also respected the integrity of the animals, and were carried out under consent and in accordance with the approved guidelines of the Brazilian Environmental Agency (License ICMBio n°31241-4).

### RNA Extraction

Following tissue extraction, the spines were immediately frozen in dry ice and conserved in RNA*later* Stabilization Reagent (QIAGEN). In order to obtain venom gland secretory material, the spines of the three specimens of each species were scraped in pool, and 100 mg of both materials were used for RNA extraction with TRIzol Reagent Purification kit (Life Technologies), according to the manufacturer’s recommendation. RNA concentration was measured using Qubit RNA Assay Kit (Life Technologies), and RNA integrity was confirmed by visualization of electrophoresed RNA on an ethidium bromide-stained agarose gel. For greater accuracy, RNA quality was assessed using an Agilent 2100 Bioanalyzer machine (Agilent Technologies) before cDNA library construction.

### cDNA library construction and sequencing

The cDNA libraries of both samples, each containing pooled RNA from three specimens, were constructed using the TruSeq RNA Sample Preparation Kit v2 (Illumina), according to the manufacturer’s protocol. Library quantity and quality was assessed using the Agilent 2100 Bioanalyzer (Agilent Technologies). Finally, both cDNA libraries were sequenced (2 × 250 bp paired-ends) on two separate Illumina MiSeq runs by the sequencing facility at the Catholic University of Brasília. After sequencing, samples were individualized according to their indexes and converted to fastq format using the MiSeq Reports (Illumina) program. Raw data from the sequencing runs were submitted to the Sequence Read Archive (SRA) repository of the National Center for Biotechnology Information (NCBI) under accession number SRR2039259.

### Sequence data pre-processing and transcriptome assembly

In order to perform quality trimming of the fastq files obtained from Illumina MiSeq sequencing, pre-processing was carried out using the Trimmomatic tool^45^ using the following parameters: sliding window:4:20; leading: 10; trailing: 10; minlen: 40. All sequences smaller than 40 bases were eliminated on the assumption that small reads might represent sequencing artifacts. Also, Trimmomatic verified the presence and removed adapter sequences that matched entries in a FASTA file containing all known Illumina adapters. Quality assessment of the Trimmomatic pre-processed data was carried out using the FastQC tool (http://www.bioinformatics.babraham.ac.uk), which confirmed that poor quality bases were removed. The resulting high quality reads were then assembled into contigs using the Trinity *de novo* assembly software^13^. In order to reduce redundancy, the program cap3^46^ was used to merge contigs with overlap length cutoff of 200 and overlap identity cutoff of 99, and contigs showing low expression (<1 FPKM) were removed. Parameters used for the Trinity assembly included a maximum memory of 30 Gigabytes, threaded on 60 processors, and a fastq sequence type. Trinity assembly, as well as all other analyses that employed high performance computers (HPCs), were done on a SGI uv100 computer system running SUSE Linux located at the Bioinformatics Laboratory of the Catholic University of Brasília.

### Transcriptome abundance estimation

In order to compute abundance estimates of the assembled transcriptome, the pre-processed high quality reads were aligned to the Trinity contigs using the Bowtie read aligner^47^. Then, based on the resulting alignments, the RNA-Seq by Expectation Maximization (RSEM) tool^14^ was used to estimate transcript abundance in terms of Fragments per kilobase of exon per million fragments mapped (FPKM).

### Transcriptome, functional and pathway annotations

#### Transcripts containing coding sequences

In order to filter out likely transcript artifacts and noise, prior to annotation, the assembled transcripts were filtered based on minimum length (> = 200 bp) and expression value (> = 1 FPKM). These transcripts were then analyzed by the TransDecoder software^15^, contained within the Trinity package^13^, which identifies candidate coding regions within transcript sequences. To detect transcript similarities with other species, the filtered transcripts were subjected to a BLASTx analysis^17^ (with e-value threshold of 10^−5^ and matching to the top hits) against the following databases: NCBI nonredundant (nr) protein sequences^16^, UniProtKB^24^ and UniProtKB/Swiss-Prot Tox-Prot^25^. The Trinotate software^15^, contained within the Trinity package^13^, was used for functional annotation, including Gene Ontology (GO) terms^48^ and presence and location of signal peptide cleavage sites (SignalP)^23^. The Web Gene Ontology Annotation Plotting (WEGO)^49^ online tool was then used for visualizing and comparing our GO annotation for both species. Based on BLASTx matches to UniProtKB sequences^24^, the Encyclopedia of Genes and Genomes (KEGG) pathway^22^ and InterPro^50^ databases were searched in order to include these annotations to the assembled transcripts.

#### Transcripts without coding sequences

Long non-coding RNAs (lncRNAs) present in the assembled transcriptomes were searched using the PLEK software^20^, which attempts to distinguish lncRNA from protein-coding sequences in high-throughput sequencing without reference genomes or annotations. This software uses a computational pipeline based on an improved *k*-mer scheme and a support vector machine algorithm to distinguish lncRNAs from protein-coding sequences^20^. The summary of the whole transcriptome analysis workflow is illustrated in Supplementary Fig. S14.

### Sequence Alignment and Phylogenetic Analysis

The highest expressed hyaluronidase transcript hits obtained from the *P. amandae* and *P. falkneri* transcriptomes were selected for alignment with a diverse set of hyaluronidase sequences obtained from other species, including venomous organisms (*Bitis arietans*, *Echis ocellatus*, *Cerastes cerastes*, *Echis pyramidum leakeyi*, *Synanceia horrida*, *Synanceia verrucosa*, *Pterois antennata* and *Pterois volitans*). The UniProtKB accession numbers of the 42 protein sequences selected for comparison are listed in Table S4. The selected sequences were aligned with the Clustal Omega Multiple Sequence Alignment software^51^ (Fig. S13), and a Maximum Likelihood phylogenetic tree based on the JTT matrix-based model^52^ was created using the Molecular Evolutionary Genetics Analysis (MEGA) software, version 6^53^. The JTT matrix-based model^52^ was selected as the best-fit model by both MEGA^53^ and ProtTest^54^ softwares. Confidence values for phylogenetic tree branching were generated by the bootstrap method (500 replicates)^55^. FigTree 1.4 was used to produce the phylogenetic tree figure.

### Molecular Modeling

For molecular modeling studies, 13 toxin transcript sequences obtained from the assembled transcriptomes of *P. amandae* and *P. falkneri* were analyzed. The families that were analyzed were the following: phospholipase A2, serine protease, cysteine-rich secretory protein (CRISP), glycoside hydrolase (hyaluronidase), flavin-containing amine oxidoreductase (L-amino acid oxidase), C-type lectin (CLEC) and vascular endothelial growth factor A (VEGFA) for both *P. amandae* and *P. falkneri*, and disintegrin (snake venom metalloproteinase) only for *P. amandae*. Phobius server^56^ was used in order to predict signal peptide and intermembrane regions, which were not considered for further analysis. Protein-protein BLAST^17^ was also carried out in order to find the best template sequences for molecular modeling. By using Modeller v 9.12^57^, 100 three-dimensional homology models were built and ranked by their free energy values. Those models with lowest free energy were then selected and validated according to their geometry, stereochemistry and energy distributions by using PROCHECK^58^. ProSA-web server^59^ was also used to calculate an overall quality score for the selected theoretical models in comparison with those scores for proteins structurally resolved by X-ray crystallography or Nuclear Magnetic Resonance (NMR) techniques.

## Additional Information

**How to cite this article**: Júnior, N. G. O. *et al.* Venom gland transcriptome analyses of two freshwater stingrays (Myliobatiformes: Potamotrygonidae) from Brazil. *Sci. Rep.* **6**, 21935; doi: 10.1038/srep21935 (2016).

## Supplementary Material

Supplementary Information

## Figures and Tables

**Figure 1:**
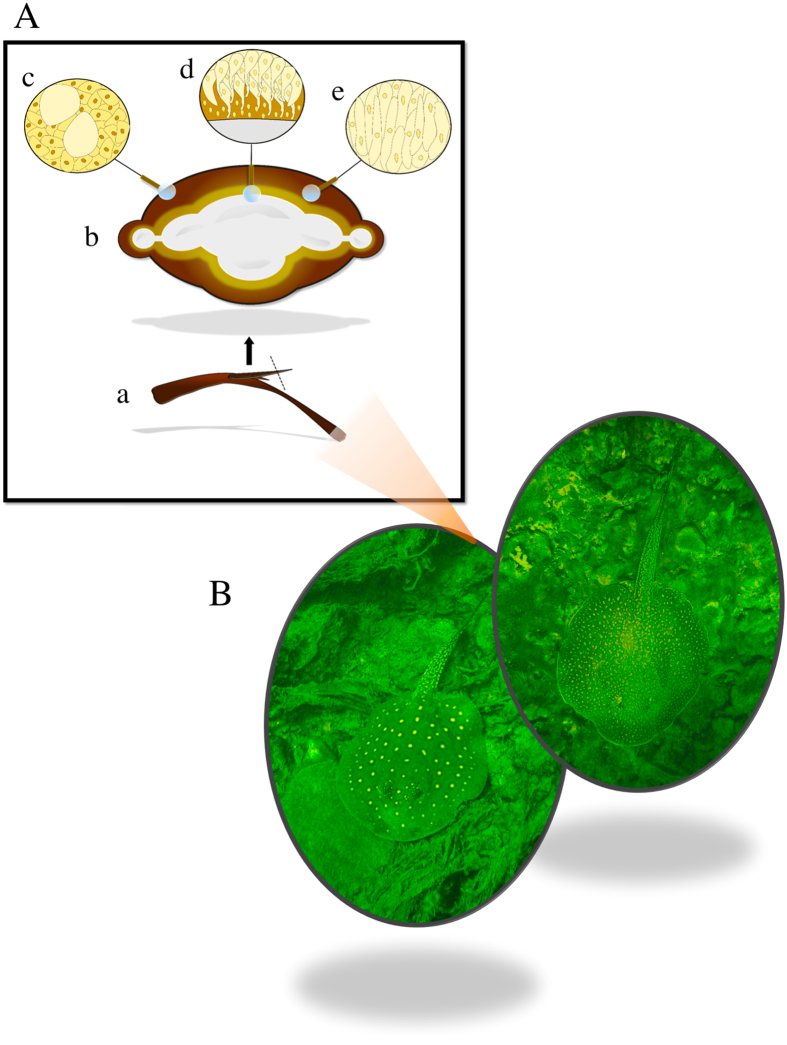
(**A**) (a) Stingray spine indicating cross section. (b) Spine cross-sectional view showing mineral region in the center and venom producing glands covering the mineral region. (c) The organization of epithelial cells in the presence of mucus producing glands. (d) Organization of venom producing cells elongated and compressed around the bone of the spine. (e) Distribution of venom producing glands. (**B**) *Potamotrygon falkneri* (left) and *P. amandae* (right) species.

**Table 1:** Summary statistics of the sequencing and the *de novo* transcriptome assembly of the *Potamotrygon amandae* and *P. falkneri* cDNA samples.

	*P. amandae*	*P. falkneri*
Before pre-processing		
Number of raw reads	14,539,142	15,375,430
Raw data (bp)	3,357,743,092	3,384,969,885
Average read length (bp)	231	220
After pre-processing		
Number of high-quality reads	13,109,527	13,730,400
Clean data (bp)	2,637,602,285	2,627,406,006
% of reads with Phred score ≥ 33	92.7	92.4
Average read length (bp)	200	207
Trinity assembly statistics		
Number of contigs	147,881	105,191
Number of contigs ≥ 1FPKM	75,083	49,219
Number of contigs ≥ 1 FPKM and containing CDSs	25,092	22,083
Contigs (bp)	50,987,297	42,504,708
N50	3,015	2,700
Average contig length (bp)	2,032	1,924
Min. contig length (bp)	293	297
Max. contig length (bp)	15,915	14,247

**Table 2:** Summary statistics of functional annotation of the *Potamotrygon amandae* and *P. falkneri* assembled transcripts.

	*P. amandae*	*P. falkneri*
Similarity Search		
Contigs with BLAST hit against NCBI nr	21,245 (84.7%)	19,316 (87.5%)
Contigs with BLAST hit against UniprotKB	17,250 (68.8%)	15,935 (72.2%)
Contigs with BLAST hit against NCBI nr and UniprotKB	17,240 (68.7%)	15,922 (72.1%)
Contigs with BLAST hits against UniprotKB Tox-Prot	426 (1.7%)	396 (1.8%)
Contigs without BLAST hits to NCBI or UniprotKB	3,837 (15.3%)	2,767 (12.5%)
Contigs with BLAST hit against *Callorhincus milii* transcriptome	15,825 (63.1%)	14,548 (65.9%)
Contigs with BLAST hit against *Latimeria chalumnae* transcriptome	17,083 (68.1%)	15,611 (70.7%)
Contigs with BLAST hit against *Danio rerio* transcriptome	17,211 (68.6%)	15,874 (71.9%)
Contigs with BLAST hit against *Potamotrygon amandae* transcriptome	—	18,423 (83.4%)
Contigs with BLAST hit against *Potamotrygon falkneri* transcriptome	20,020 (79.8%)	—
Functional Annotation		
Contigs with Gene Ontology terms assigned	16,921 (64.9%)	15,394 (69.7%)
Contigs with PFAM terms assigned	18,130 (72.3%)	16,387 (74.2%)
Contigs with InterPro terms assigned	16,961 (67.6%)	15,666 (70.9%)
Contigs with KEGG terms assigned	14,131 (56.3%)	13,147 (59.5%)
Contigs with signal peptide (SignalP 4.1) hits	1,441 (5.1%)	1,185 (4.9%)
Long non-coding RNA (lncRNA) hits in contigs without CDS (PLEK)	1,913 (3.8%)	1,611 (5.9%)

**Table 3:** Top 25 most abundant *Potamotrygon amandae* transcript hits against UniProtKB Tox-Prot.

*P. amandae*transcript id	UniprotAccession	FPKM	Protein Description
TR15727|c7_g3_i1	J3S820	22201.33	Hyaluronidase (Venom spreading factor)
TR46859|c3_g1_i1	U3EQ60	1225.86	Translationally-controlled tumor protein homolog (TCTP)
TR14142|c0_g2_i1	Q8JI38	891.77	Cysteine-rich venom protein latisemin (CRVP)
TR14142|c3_g1_i1	P81656	854.22	Venom allergen 5 (Antigen 5) (Ag5) (Cysteine-rich venom protein) (CRVP) (allergen Pol d 5)
TR13715|c2_g1_i1	Q8AY75	835.98	Calglandulin
TR17445|c0_g2_i1	J3SE80	629.15	Cystatin-2
TR46921|c0_g1_i1	Q5R231	401.11	Hemolytic toxin Avt-1 (Avt-I)
TR46991|c0_g1_i1	B2BS84	354.6	Putative Kunitz-type serine protease inhibitor
TR61475|c0_g1_i1	P0CV91	304.79	Peroxiredoxin-4
TR8697|c0_g1_i1	Q8T0W5	211.07	Cysteine-rich venom protein 1
TR12644|c0_g3_i2	Q02989	206.6	Alpha-latroinsectotoxin-Lt1a
TR73112|c0_g1_i1	Q6T269	141.02	Kunitz-type serine protease inhibitor bitisilin-3
TR5350|c0_g2_i1	C0HJF3	121.14	Analgesic polypeptide HC3 (APHC3)
TR5350|c0_g1_i1	P68425	117.78	Kunitz-type serine protease inhibitor kappa-theraphotoxin-Hh1a
TR16987|c4_g1_i3	Q58L93	104.65	Venom prothrombin activator porpharin-D
TR36287|c0_g1_i1	A8QL49	101.89	Zinc metalloproteinase-disintegrin-like BmMP (Snake venom metalloproteinase)
TR11241|c0_g1_i1	Q9DF56	100.95	Acidic phospholipase A2 (Phosphatidylcholine 2-acylhydrolase)
TR15369|c0_g2_i1	Q2XXL4	95.12	Vespryn
TR13307|c0_g2_i1	G4V4G1	93.55	Insulin-like growth factor-binding protein-related protein 1 (IGFBP-rP1)
TR11355|c0_g2_i1	Q25338	87.69	Delta-latroinsectotoxin-Lt1a
TR15061|c1_g1_i1	P83234	86.84	Ohanin
TR15657|c0_g1_i1	A6MFK8	71.33	Venom prothrombin activator vestarin-D2
TR75353|c0_g1_i1	Q76B45	70.84	Blarina toxin
TR4772|c0_g1_i1	Q7M4I3	59.88	Venom protease

**Table 4:** Top 25 most abundant *Potamotrygon falkneri* transcript hits against UniProtKB Tox-Prot.

*P. falkneri*transcript id	UniprotAccession	FPKM	Protein Description
TR10580|c4_g1_i1	J3S820	9488.18	Hyaluronidase (Venom spreading factor)
TR15149|c0_g1_i1	Q5R231	757.19	Hemolytic toxin Avt-1 (Avt-I)
TR43499|c1_g1_i1	U3EQ60	734.38	Translationally-controlled tumor protein homolog (TCTP)
TR3678|c0_g1_i1	Q3SB11	644.06	Calglandulin
TR49323|c1_g1_i1	B2BS84	450.57	Putative Kunitz-type serine protease inhibitor
TR44732|c0_g1_i1	P0CV91	397.49	Peroxiredoxin-4
TR232|c1_g1_i2	Q8T0W5	386.31	Cysteine-rich venom protein 1 (cvp1)
TR3629|c1_g2_i1	P81656	357.2	Venom allergen 5 (Antigen 5) (Ag5) (Cysteine-rich venom protein) (CRVP)
TR3414|c0_g1_i1	Q3SB11	318.87	Calglandulin
TR3629|c0_g2_i1	Q8JI38	299.5	Cysteine-rich venom protein latisemin (CRVP)
TR11273|c0_g3_i1	Q2XXL4	172.92	Vespryn
TR9744|c0_g2_i1	Q8MQS8	156.83	Venom serine protease 34 (allergen Api m 7)
TR44139|c0_g1_i1	Q8AY75	150.86	Calglandulin
TR14942|c8_g1_i1	Q9XZC0	150.43	Alpha-latrocrustotoxin-Lt1a (Alpha-LCT-Lt1a)
TR12189|c0_g1_i1	G0LXV8	150.2	Alpha-latrotoxin-Lh1a
TR43799|c0_g1_i1	A8QL49	143.04	Zinc metalloproteinase-disintegrin-like BmMP (Snake venom metalloproteinase)
TR14826|c2_g1_i1	J3SE80	140.7	Cystatin-2
TR125|c0_g1_i1	Q7M4I3	118.09	Venom protease (allergen Bom p 4)
TR20621|c0_g1_i1	Q02989	114.49	Alpha-latroinsectotoxin-Lt1a (Alpha-LIT-Lt1a)
TR8110|c0_g1_i1	P0DJ69	108.68	Kunitz-type serine protease inhibitor HNTX-852
TR14758|c9_g3_i3	Q58L93	107.52	Venom prothrombin activator porpharin-D
TR8110|c0_g1_i2	C0HJF3	106.37	Analgesic polypeptide HC3
TR6103|c0_g1_i3	A8YPR9	86.64	Snake venom metalloprotease inhibitor
TR38838|c0_g1_i1	Q6T269	81.19	Kunitz-type serine protease inhibitor bitisilin-3
