# Supplementary material for: Venom gland transcriptome analyses of two freshwater stingrays (Myliobatiformes: Potamotrygonidae) from Brazil
Source: Sci Rep. 2016 Feb 26;6:21935. doi: 10.1038/srep21935 (PMC4768133; doi:10.1038/srep21935)
Supplement: Supplementary Information [file srep21935-s1.pdf]

Supplementary Information

Venom gland transcriptome analyses of two freshwater stingrays (Myliobatiformes: Potamotrygonidae) from Brazil

Nelson Gomes de Oliveira Júnior, Gabriel da Rocha Fernandes, Marlon Henrique Cardoso, Fabrício F. Costa, Elizabete de Souza Cândido, Domingos Garrone Neto, Márcia Renata Mortari, Elisabeth Ferroni Schwartz, Octávio Luiz Franco, Sérgio Amorim de Alencar

Supplementary Figures:

Figure S1: Transcript length histograms of the *P. amandae* and *P. falkneri* Trinity-assembled contigs.

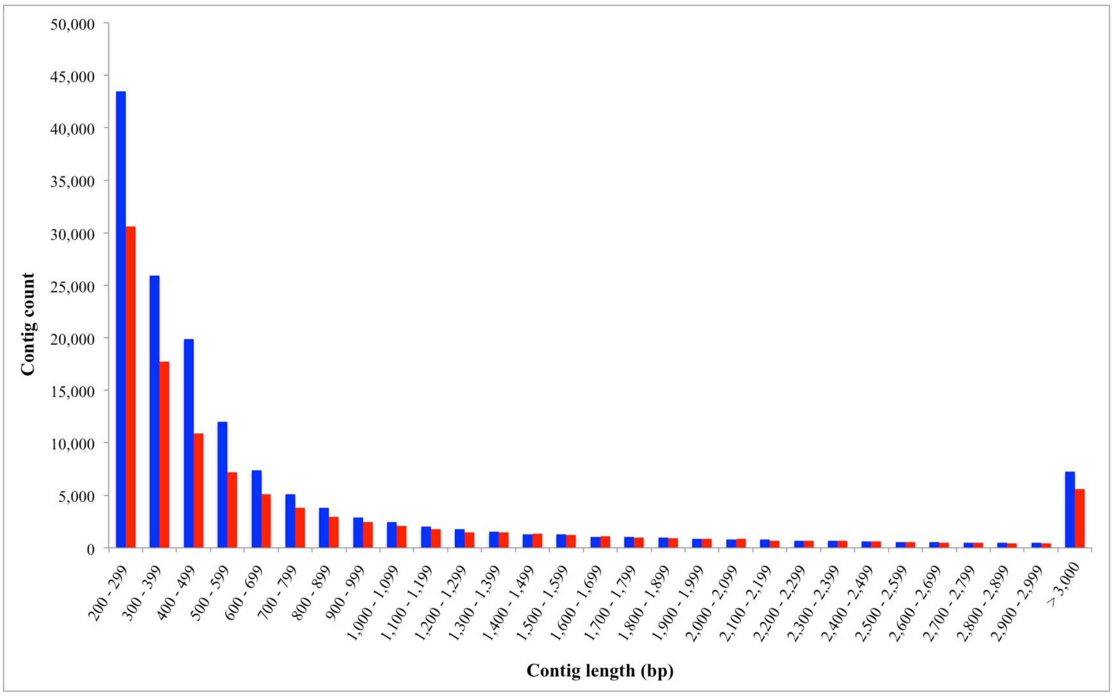

The length distribution of the *P. amandae* (blue) and *P. falkneri* (red) contigs reveals that a major proportion of the assembled contigs range between 200 to 599 bp in length.

Figure S2: E-value distribution of the top BLASTx hits.

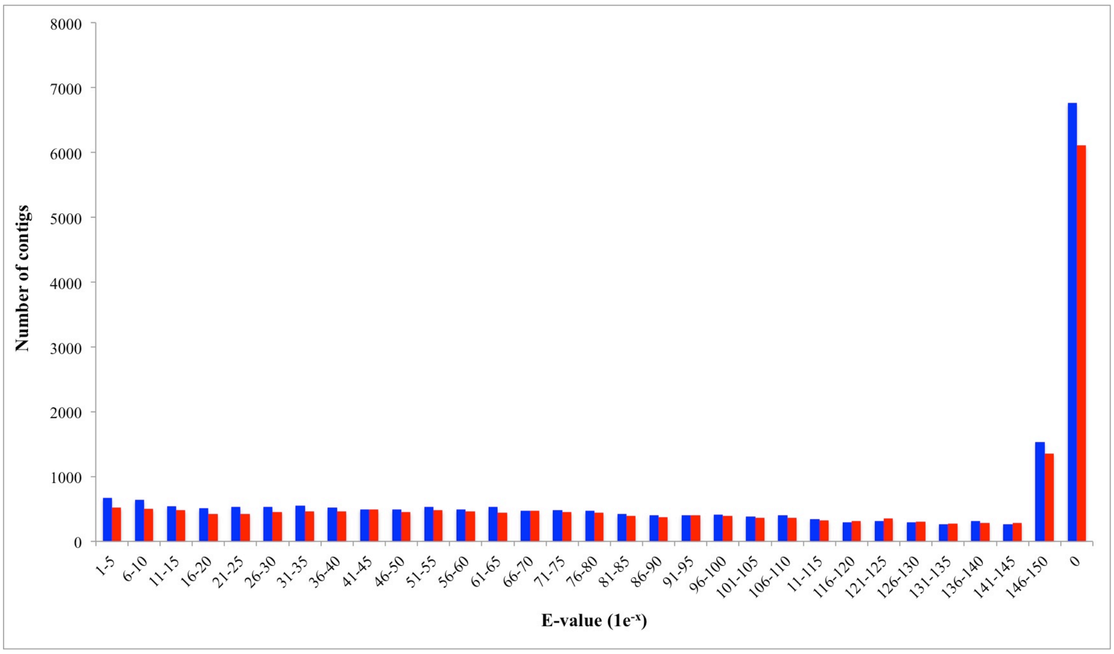

Sequences with e-value equal to 0 are represented in the right peak. The cut-off used was $1e^{-5}$. *P. amandae* and *P. falkneri* contigs are represented in blue and red, respectively.

Figure S3: Lysine degradation pathway.

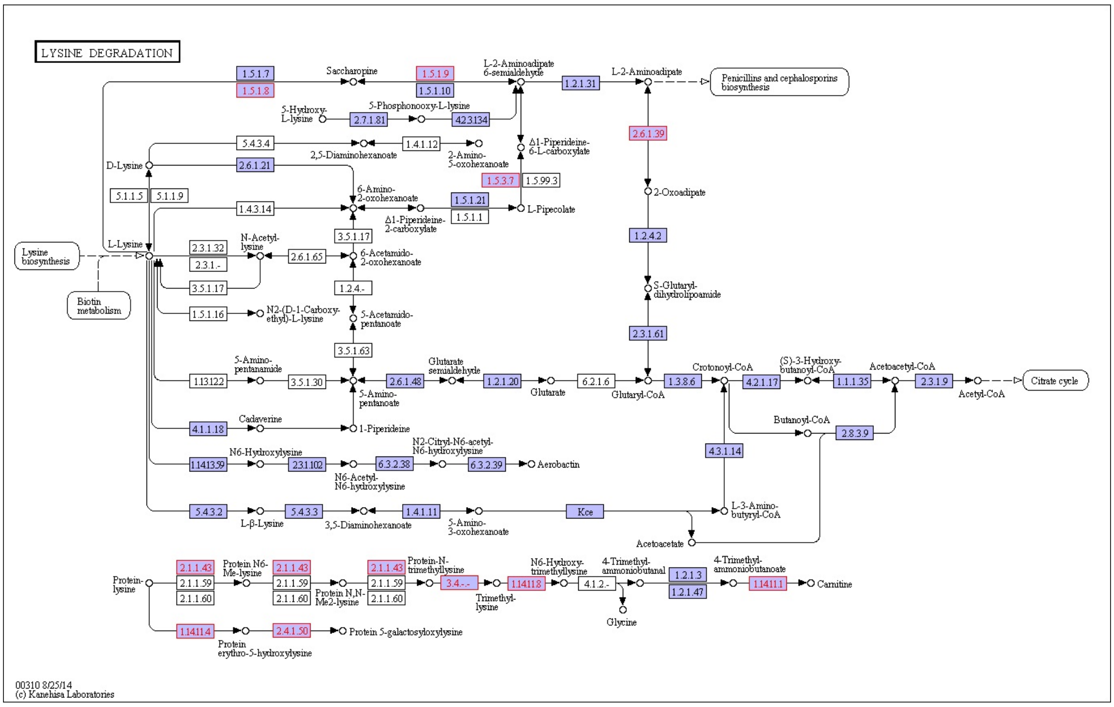

Figure S4: Arginine and proline metabolism pathway.

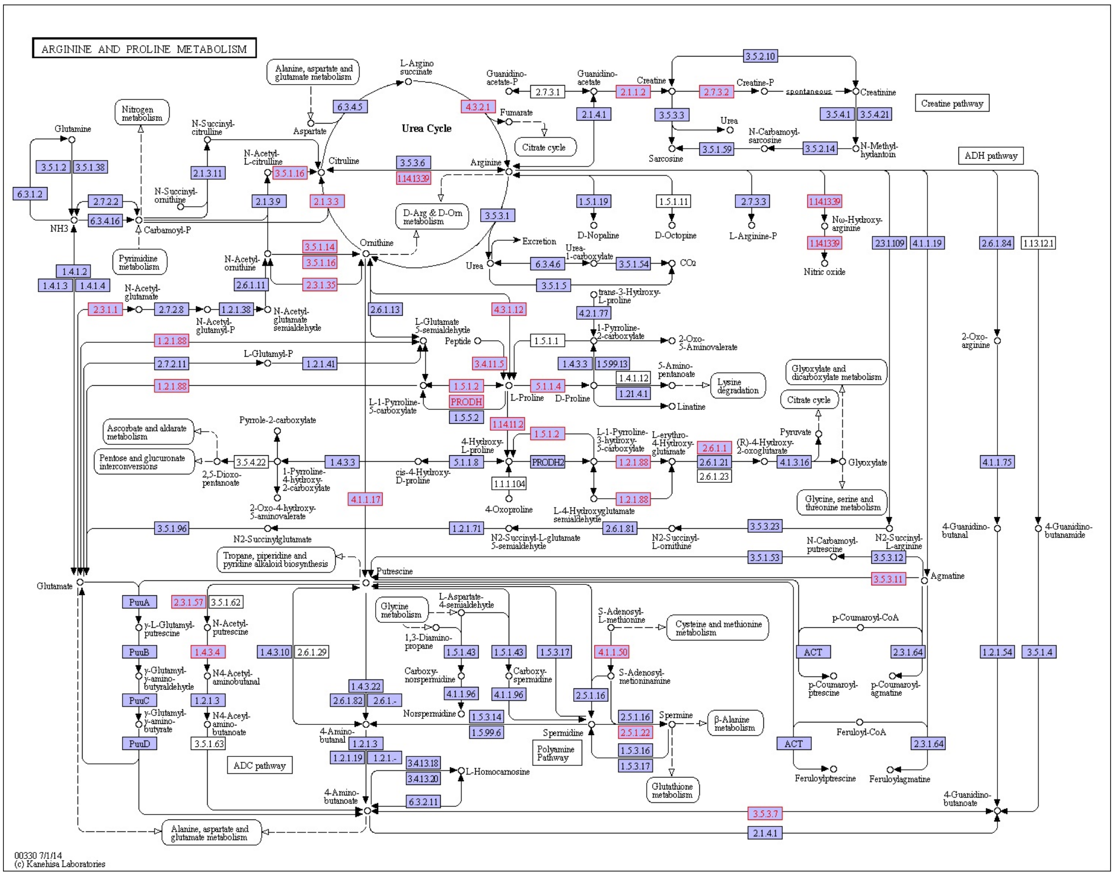

Figure S5: Cysteine and methionine metabolism pathway.

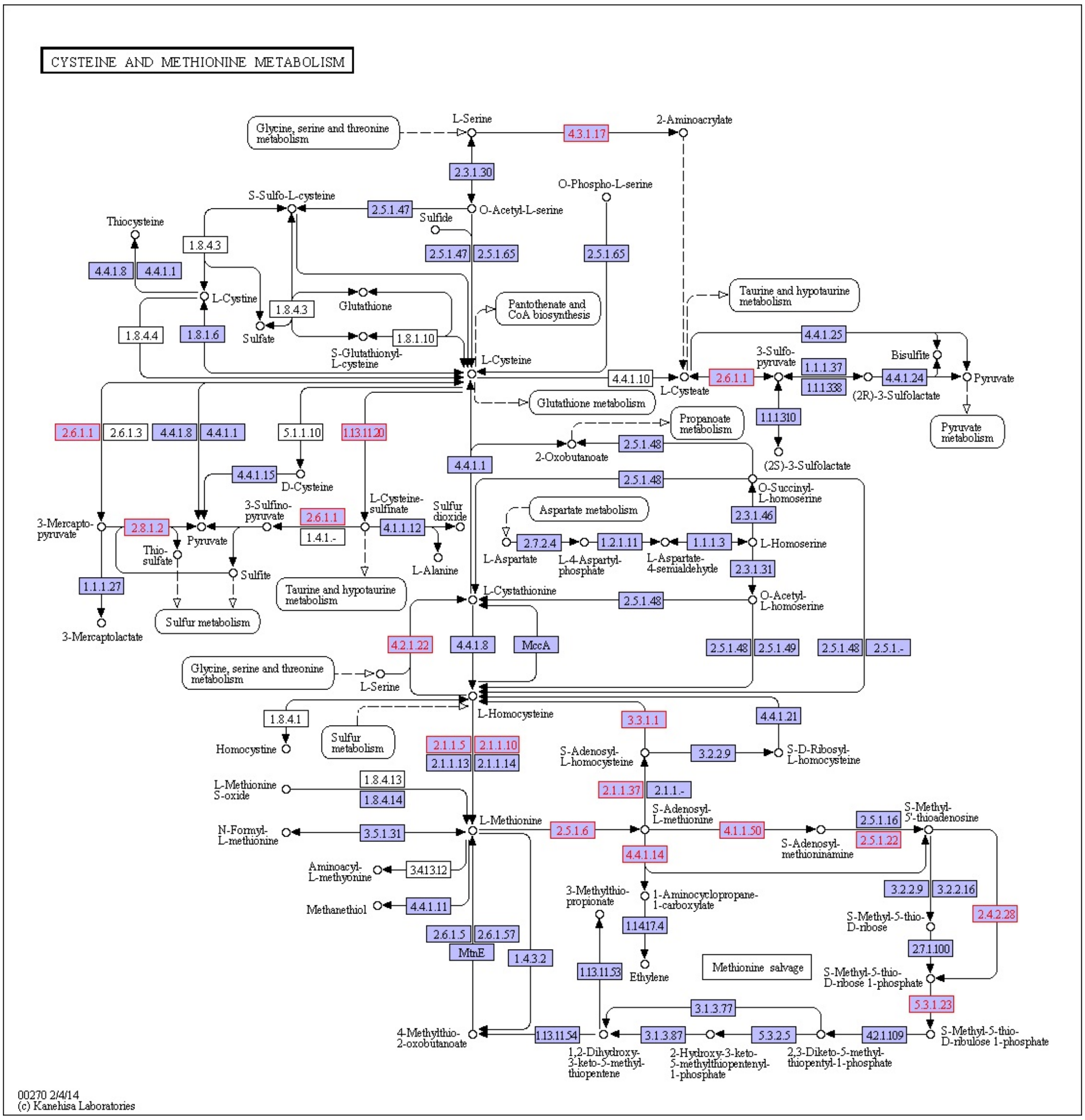

Figure S6: Glycine, serine and threonine metabolism pathway.

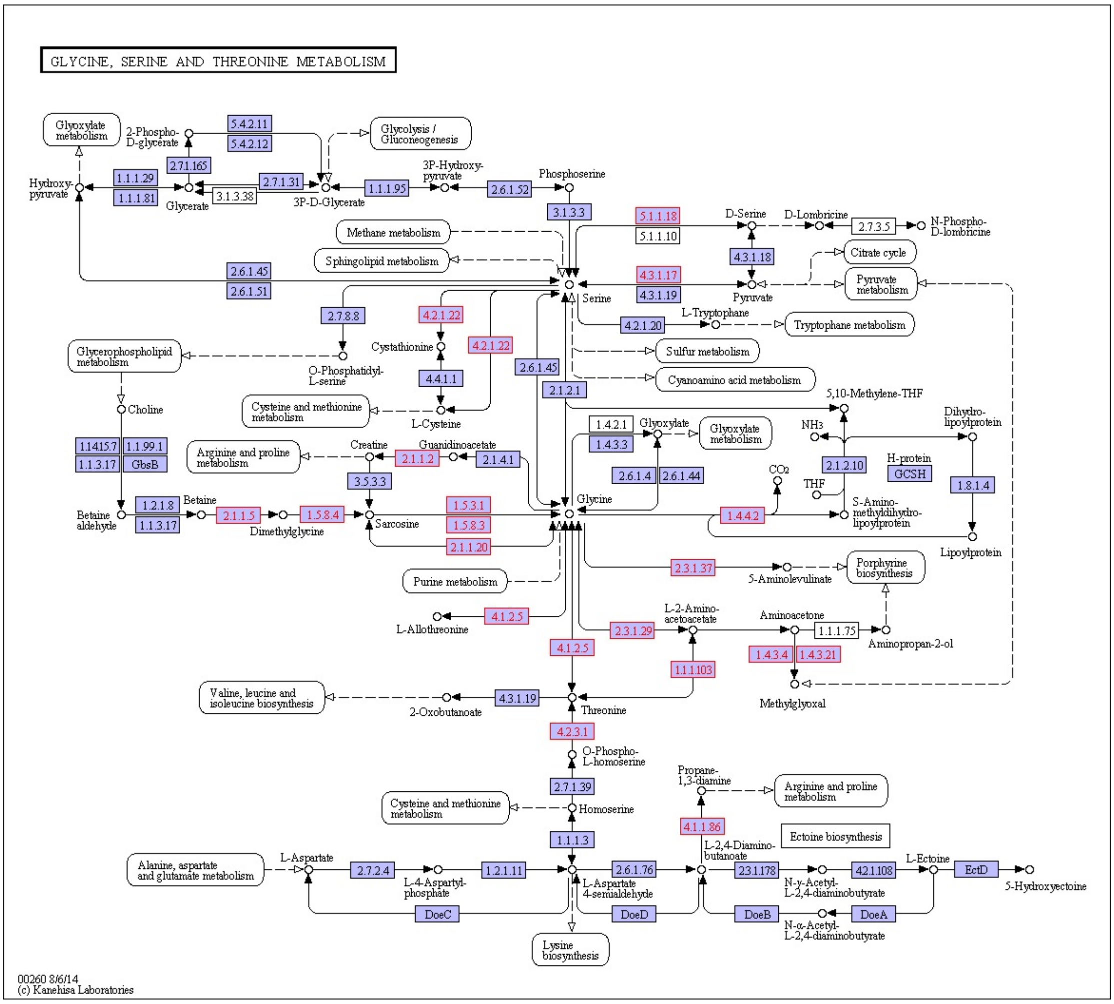

Figure S7: Citrate cycle (TCA cycle) pathway.

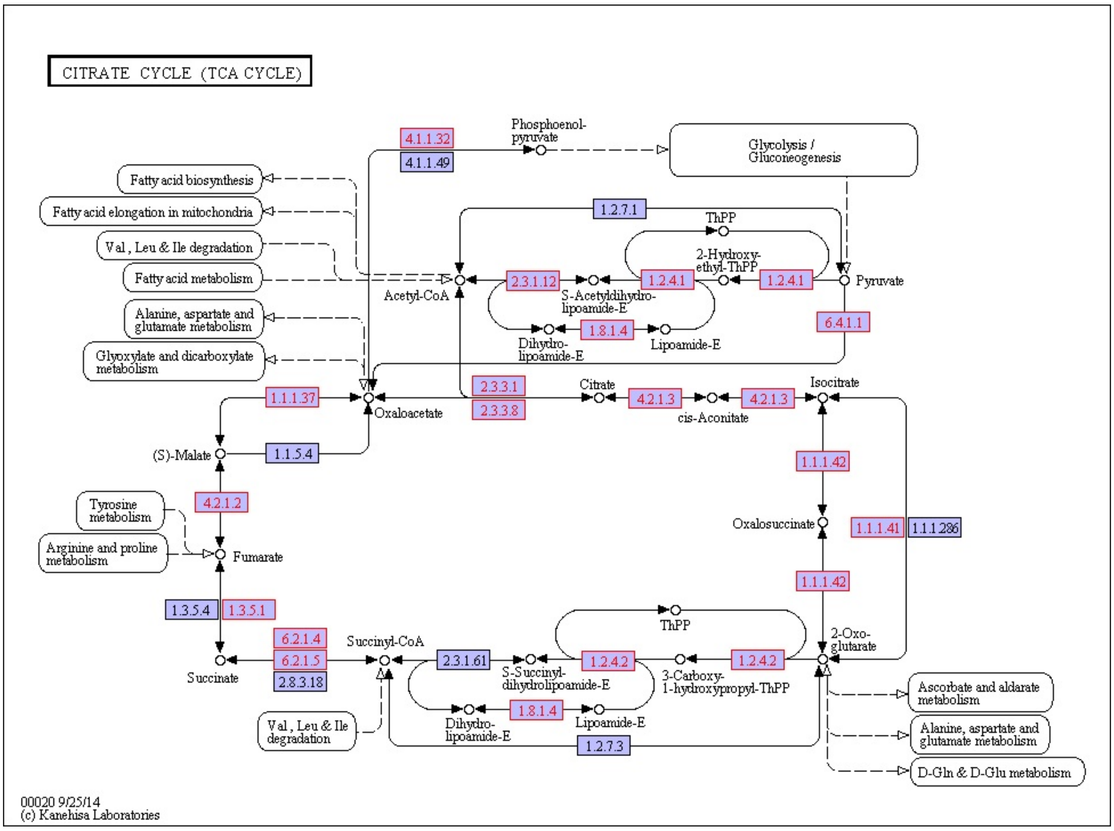

Figure S8: Glycolysis/Gluconeogenesis pathway.

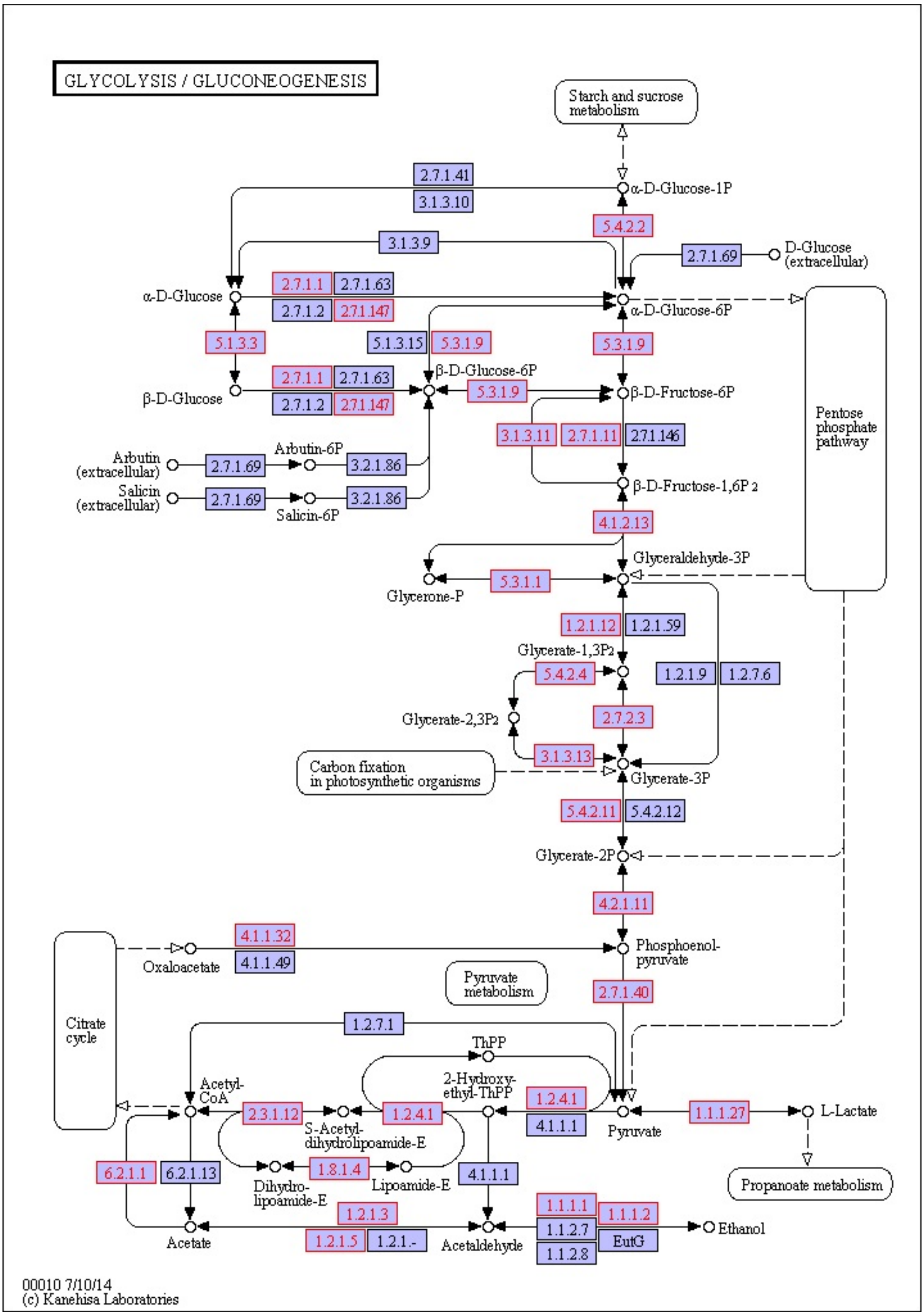

Figure S9: Pyruvate metabolism pathway.

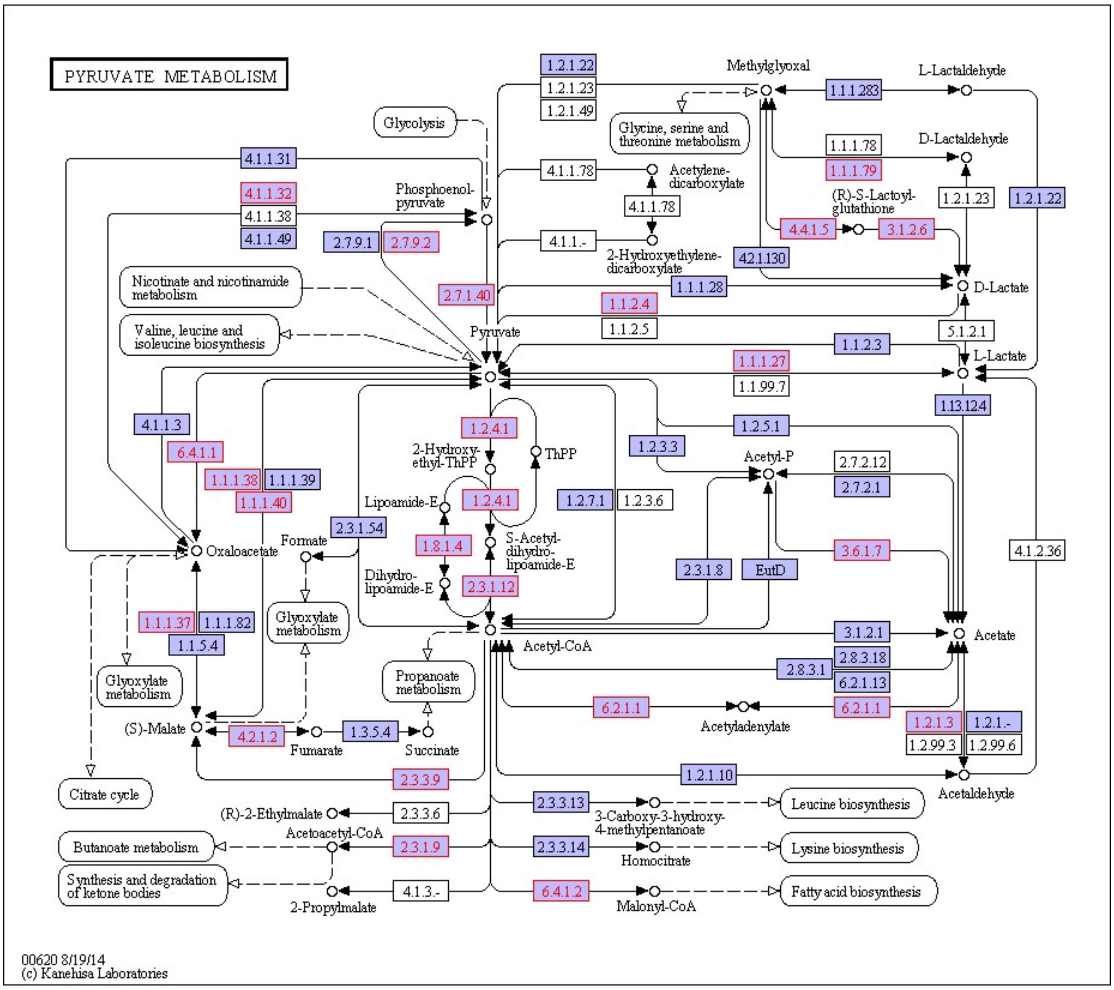

Figure S10: Fatty acid degradation pathway.

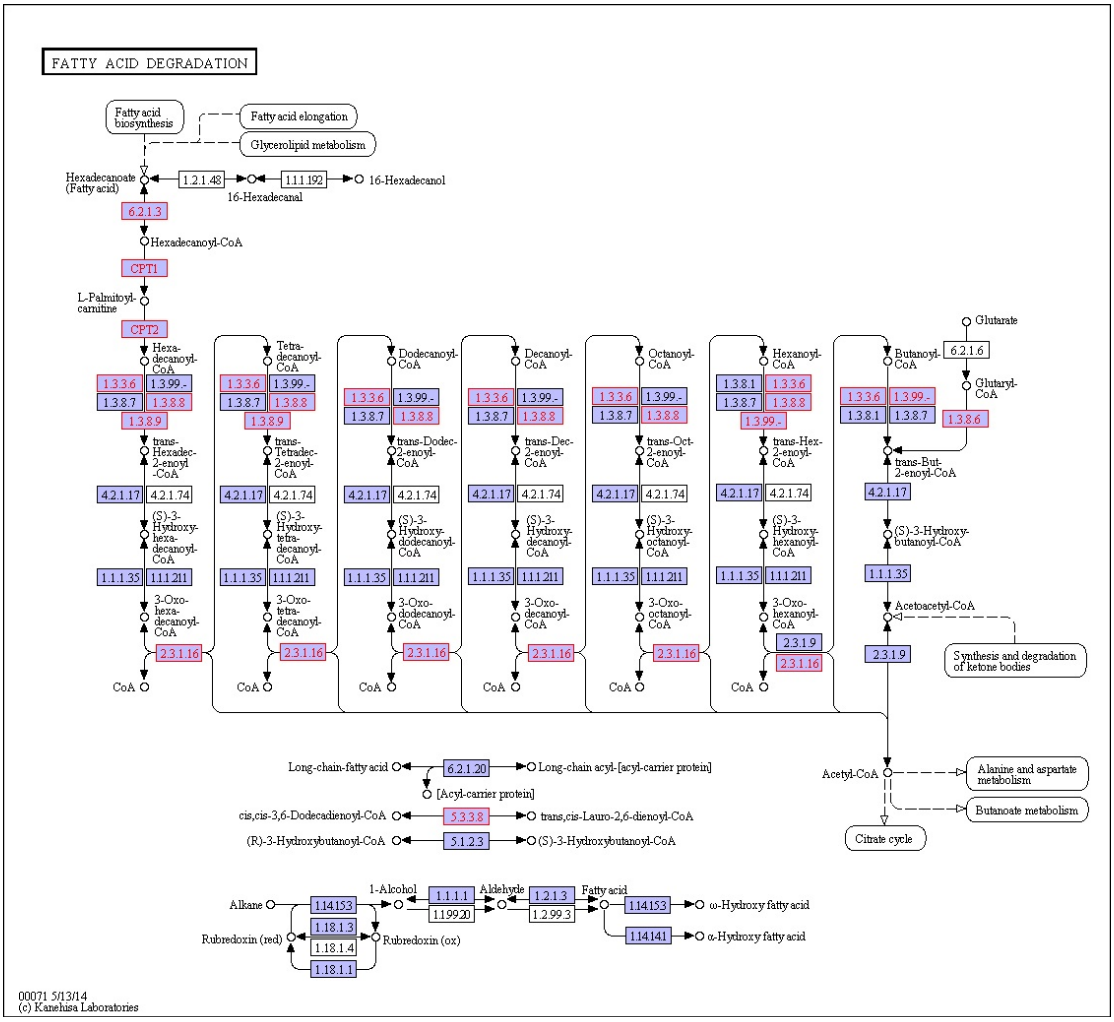

Figure S11: Sphingolipid metabolism pathway.

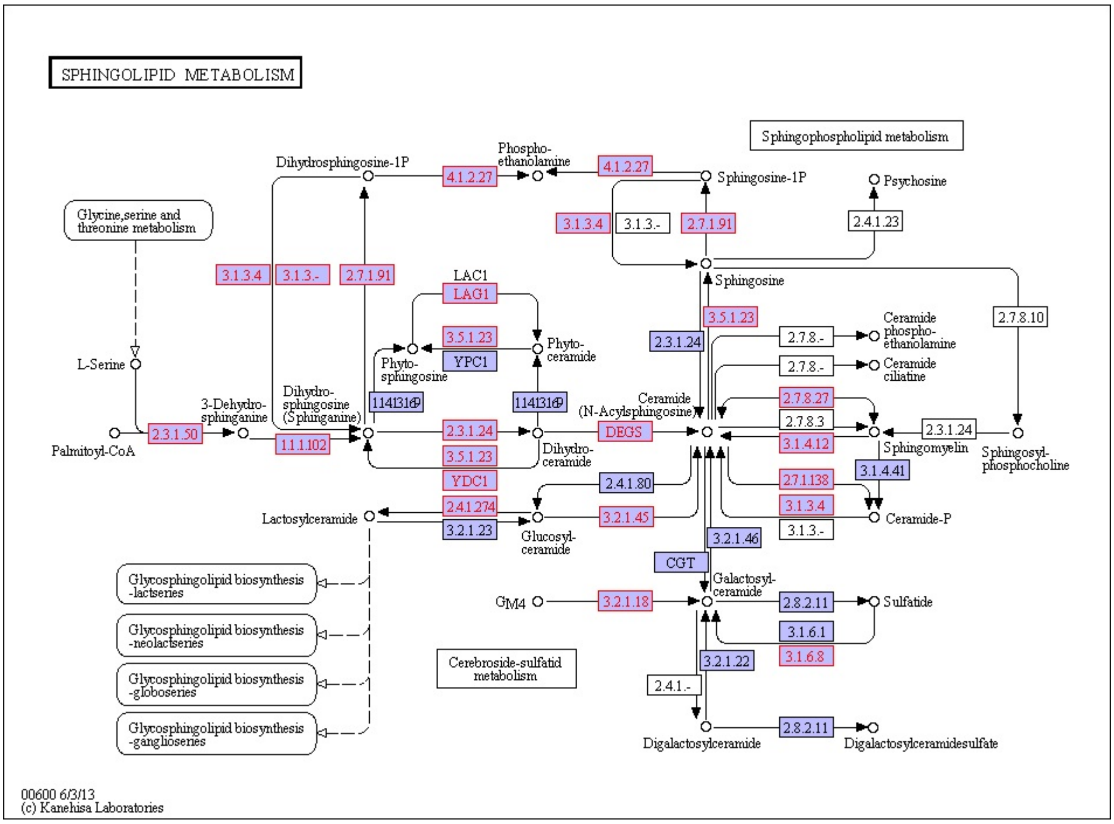

Figure S12: Glycerophospholipid metabolism pathway.

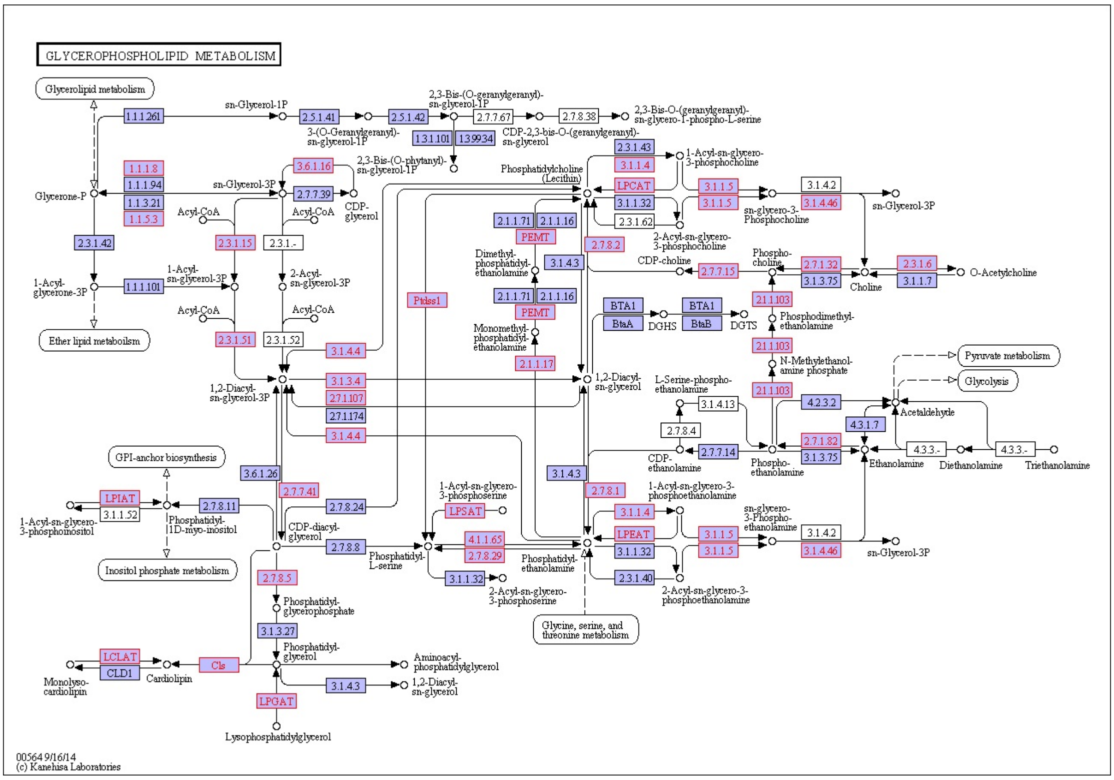

Figure S13: Clustal Omega alignment of hyaluronidase proteins from a diverse set of organisms.

```
EOB06026_Anas_platyrhynchos                 ------------------------------------------------------------
EMC77798_Columba_livia                      ------------------------------------------------------------
XP_002190553_Taeniopygia_guttata            -------------------------------------------------METVRLQSFGI
BAM66299_Pterois_antennata                  -------------------------------------------------MNHLKLLDDQH
BAM66300_Pterois_volitans                   -------------------------------------------------MNHLKLLDDQH
AAO74499_Synanceia_horrida                  -------------------------------------------------MIKLKFLYVQH
BAJ54082_Synanceia_verrucosa                -------------------------------------------------MNQVKFLDVQH
NP_001094250_Rattus_norvegicus              ----------------------------------------------------MQLLSEGQ
XP_001370718_Monodelphis_domestica          ----------------------------------------------------MKTLSERR
XP_001502439_Equus_caballus                 ----------------------------------------------------MKLLSEGQ
XP_002752104_Callithrix_jacchus             ----------------------------------------------------MKVLSEGQ
XP_002818436_Pongo_abelii                   ----------------------------------------------------MKVLSEGQ
AAI04791_Homo_sapiens                       ----------------------------------------------------MKVLSEGQ
XP_527872_Pan_troglodytes                   ----------------------------------------------------MKVLSERQ
XP_003217668_Anolis_carolinensis            ------------------------------------------------------------
ABI33944_Bitis_arietans                     ------------------------------------------------------------
ABI33937_Echis_ocellatus                    ------------------------------------------------------------
ABI33938_Cerastes_cerastes                  ------------------------------------------------------------
ABI33941_Echis_pyramidum_leakeyi            ------------------------------------------------------------
TR15727|c7_g3_i1_Potamotrygon_amandae       ------------------------------------------------------------
TR10580|c4_g1_i1_Potamotrygon_falkneri      ------------------------------------------------------------
XP_007896746_Callorhinchus_milii            -----------MKPEAPL-------------SLQRTSRHSFLEDKEFTANGNCTMSS---
XP_006012182_Latimeria_chalumnae            ------------------------------------------------------------
XP_009685663_Struthio_camelus_australis     ------------------------------------------------------------
XP_010213407_Tinamus_guttatus               ------------------------------------------------------------
XP_008943867_Merops_nubicus                 ------------------------------------------------------------
XP_013054655_Anser_cygnoides_domesticus     ------------------------------------------------------------
NP_001295181_Gallus_gallus                  ------------------------------------------------------------
XP_006024588_Alligator_sinensis             ------------------------------------------------------------
XP_006268718_Alligator_mississippiensis     ------------------------------------------------------------
XP_005305767_Chrysemys_picta_bellii         ------------------------------------------------------------
EMP32607_Chelonia_mydas                     ------------------------------------------------------------
XP_006122657_Pelodiscus_sinensis            ------------------------------------------------------------
XP_006633469_Lepisosteus_oculatus           MTGRRRQGAGGVDRQGGVTMEIWCSGGVTKANKSKESRQEIKGEVQKSKTGETSVIN---
XP_012678206_Clupea_harengus                ------------------------------------------------------------
NP_001074140_Danio_rerio_                   ------------------------------------------------------------
XP_007246617_Astyanax_mexicanus             ------------------------------------------------------------
XP_005810535_Xiphophorus_maculatus          ------------------------------------------------------------
XP_003449275_Oreochromis_niloticus          ------------------------------------------------------------
XP_003967307_Takifugu_rubripes              ------------------------------------------------------------
ACI32917_Salmo_salar                        ----------------------------------------------------MGVAP---
NP_001119986_Xenopus_tropicalis             -------------------------------------------------------MP---
```

```
EOB06026_Anas_platyrhynchos               ----------------ASGMVFATVLVSCCS--SLNIRARPLISNSPFLSIWNAPTELCS
EMC77798_Columba_livia                    ----------------ASSMVFATLLVSCCS--SLNIRARPLFSNLPFLSIWNAPTELCT
XP_002190553_Taeniopygia_guttata          CVTCTYP--------VASGVVFATLLVSCCS--SLNIRARPLLSNSPFLSIWNAPTELCT
BAM66299_Pterois_antennata                FF-----------------SIFIPMLALMWSGWALPRTDPPLCPGHHFMFMWNAPTELCE
BAM66300_Pterois_volitans                 FF-----------------SIFIPMLALMWSGWALPRTDPPLCPGHHFMFMWNAPTELCE
AAO74499_Synanceia_horrida                FF-----------------SISVTMMSLLRSGGALPWTDPPLHPGHPFLFTWNAPTELCG
BAJ54082_Synanceia_verrucosa              FF-----------------SITVTMISLLCSGWALPWTDPPLRPSHPFLFTWNAPTELCD
NP_001094250_Rattus_norvegicus            LRRCVVQPVHLTS---GLLI--F---FILKSISSLKPARLPVYQRKPFIAAWNAPTDLCL
XP_001370718_Monodelphis_domestica        LILHIVQPSYLTS---WLLV--S---FILKPIASLKPAQLPIYQRKPFIAAWNAPTDQCS
XP_001502439_Equus_caballus               FRFCVVQPIHLTS---WLLI--F---FILKSISSLKPARLPIYQRKPFIAAWNAPTDQCL
XP_002752104_Callithrix_jacchus           LRLCVVQPVHLTS---WLLI--F---FILKSISCLKPARLPIYQRKPFIAAWNAPTDQCL
XP_002818436_Pongo_abelii                 LRICVVQPVHLTS---WLLI--F---FILKSISCLKPARLPIYQRKPFIAAWNAPTDQCL
AAI04791_Homo_sapiens                     LKLCVVQPVHLTS---WLLI--F---FILKSISCLKPARLPIYQRKPFIAAWNAPTDQCL
XP_527872_Pan_troglodytes                 LKLCVVQPVHLTS---WLLI--F---FILKSISCLKPARLPIYERKPFIAAWNAPTDQCL
XP_003217668_Anolis_carolinensis          ---------MLMIMADWTGSWAFFLSVFMTPWDFGVARKGPAILHHSFITVWNAPSQACQ
ABI33944_Bitis_arietans                   ---------MYHLWIKCLAAWIF---LKRCNGVHAMPAKAPMYPNEPFIVLWNAPTTQCP
ABI33937_Echis_ocellatus                  ---------MYHLWIKCLAAWIF---LKRFNGVHVMHAKAPMYPNEPFLVFWNAPTTQCR
ABI33938_Cerastes_cerastes                ---------MYHIWIKFLAAWIF---LKRFNGVHVMQAKAPMYRNEPFLVFWNAPTTQCR
ABI33941_Echis_pyramidum_leakeyi          ---------MYHIWIKFLAAWIF---LKRFNGVHVMQAKAPMYRNEPFLVFWNAPTTQCR
TR15727|c7_g3_i1_Potamotrygon_amandae     LNIMSVTNISKNIFGRLLITAII---MCLSSGQYFKQAKSPVLPDNPFIVVWNAPTESCK
TR10580|c4_g1_i1_Potamotrygon_falkneri    ---MSATNISKNIFGRLLITAII---MCLSSGQYFKQAKSPVLPDNPFIVVWNAPTESCK
XP_007896746_Callorhinchus_milii          T--CIPRKMFTKQLSSATALLIV---LQIACGQEFKQAKVPVLPYKPFIVVWNAPTEQCR
XP_006012182_Latimeria_chalumnae          ---MKATG--KKVLKIGLAVMFL---FTLGSSQQVKQAKSPVLPDKPFIVVWNAPTEQCR
XP_009685663_Struthio_camelus_australis   ----------MKVWVKWMAISVL---LTMVCGQLLKQAKAPLLLHKPFIVVWNAPTEQCR
XP_010213407_Tinamus_guttatus             ----------MDVWVKCTAVSVL---LTAVCGRWLKQAKAPLLLHKPFIVVWNAPTEQCR
XP_008943867_Merops_nubicus               ----------MDVWVKLLAVTVL---LTVVDAQLPKPARAPLVLHKPFVVVWNAPTEQCR
XP_013054655_Anser_cygnoides_domesticus   ----------MDLWIKRLAVTAL---LAVAGAQLRKQAQAPLLLHKPFIVVWNAPTEQCR
NP_001295181_Gallus_gallus                ----------MDLWIRWLAVTVL---LTMGDEQLPKQAQAPLLLHKPFVVVWNAPTEQCR
XP_006024588_Alligator_sinensis           ---------MCNLWIKWIAITML---LIMTNGQLLKQAKAPLLLHKPFIVVWNAPTEQCR
XP_006268718_Alligator_mississippiensis   ---------MCNLWIKWIAITML---LIMTNGQLLKQAKAPLLLHKPFIVVWNAPTEQCR
XP_005305767_Chrysemys_picta_bellii       ---------MCNLWIKWIEITML---LIMVDGQLLKQAKAPLLLHKPFIVVWNAPTEQCR
EMP32607_Chelonia_mydas                   ---------MCNLWIKWIEITML---LIMVDGQLFKQAKAPLLLHKPFIVVWNAPTEQCR
XP_006122657_Pelodiscus_sinensis          ---------MCSLWSQWIAVTAL---FIMVDGQMLKQAQAPLLLHKPFLVVWNAPTEQCR
XP_006633469_Lepisosteus_oculatus         VDLHAMSAVCPTLFICWRAFMFF---IWHVACQQFKQAKIPLLQHKPFIVVWNAPTESCR
XP_012678206_Clupea_harengus              ---MRPTG-CWVLVCLCA----A---HVGVLAEQLKQASTPLLPHRPFVVVWNAPTESCR
NP_001074140_Danio_rerio_                 ---MALIG--FLVVAVC-----G---LLVAWADQLKQARTPLLPHRPFVVVWNAPTESCR
XP_007246617_Astyanax_mexicanus           ---MAVSD--LLTVVLW-----G---LVVVQGEQLKQAHTPLLPHRPFVVVWNAPTESCR
XP_005810535_Xiphophorus_maculatus        ---MVQIGLHLLPLVLWLGT-EE---KLQVQGHRTKPAQTPLIPHLPFVIIWNAPTESCR
XP_003449275_Oreochromis_niloticus        ---MVLMALHLLLLVLWVCI---------VDGDQTKPTQPPLIPHKPFVVVWNAPTESCR
XP_003967307_Takifugu_rubripes            ----------------------------------MKPARAPLIPHRPFVVVWNAPTESCR
ACI32917_Salmo_salar                      HSLLALTG--QLPWLLLLALLTP---WNVLRAEELKPTRWPMFSQKPLLLAWNAPTEDCR
NP_001119986_Xenopus_tropicalis           C-CLSFLP--AFLWLFLGAAA-N---GQLSDSWLNKPTFRPVFTRRPFIIAWNAPTQDCP
                                                                                  *      ::  ****:  *

EOB06026_Anas_platyrhynchos               ERTGVHLDMTFFSLIGSTLKTSIGQNITLFYPDRLGYYPYINEITGEAFNGGLPQLLQLE
EMC77798_Columba_livia                    EKAGVQLDMNFFSLTGSTLKTSIGQNITLFYPDRLGYYPYKNEVTGEAFNGGLPQLSLLE
XP_002190553_Taeniopygia_guttata          ERTGVQLDMNFFPLIGSTLKTSVGQNITLFYPDRIGYYPYKNEITGEAFNGGLPQLSLLE
BAM66299_Pterois_antennata                TRFSMPLDLSYFQLISSTLKTATNQTISLFYIDRFGIFPHINEKTGKIYDKGLPQLIVMQ
BAM66300_Pterois_volitans                 TRFSMPLDLSYFQLISSTLKTATNQTISLFYIDRFGIFPHINEKTGKIYDKGLPQLIDMQ
AAO74499_Synanceia_horrida                IRFGMPLDLSYFDFVSSTLKSATNQSISIFYTDRFGVFPYVNEKTGKMYNGGLPQLIDLE
BAJ54082_Synanceia_verrucosa              IRFGMPLDLSYFDFVSSTLKSATNQSISIFYTDRFGVFPYVNEKTGKMYNGGLPQLIDLE
NP_001094250_Rattus_norvegicus            IKYNLAVNLKVFQMVGSPRLKDRRQNVVIFYANKLGSYPWYTS-EGVPINGGLPQNTSLQ
XP_001370718_Monodelphis_domestica        TKYNIMLNLKMFHIIGSPLAKARGQNVTIFYVNRLGYYPWYTS-QGVPINGGLPQNFSLQ
XP_001502439_Equus_caballus               IKYNIRLNLKMFQVIGSPLAKARGQNVTIFYVNRLGYYPWYTS-QGVPVNGGLPQNISLQ
XP_002752104_Callithrix_jacchus           IKYNLRLNLKMFQVIGSPLAKARGQNVTIFYVNRLGYYPWYTS-QGVPINGGLPQNVSLQ
XP_002818436_Pongo_abelii                 IKYNLRLNLKIFPVIGSPLAKARGQNVTIFYVNRLGYYPWYTS-QGVPINGGLPQNISLQ
AAI04791_Homo_sapiens                     IKYNLRLNLKMFPVIGSPLAKARGQNVTIFYVNRLGYYPWYTS-QGVPINGGLPQNISLQ
XP_527872_Pan_troglodytes                 IKYNLRLNLKMFPVIGSPLAKARGQHVTIFYVNRLGYYPWYTS-QGVPINGGLPQNISLQ
XP_003217668_Anolis_carolinensis          EKYNVSLDLGIFDVVLNQNEFFSGEDITLFYSNRLGYYPYYTE-DGFPVFGGLPQNASLK
ABI33944_Bitis_arietans                   LRYKVDLDLKTFHIVANPNDSLSGSVVTIFYPNHLGVYPHIDE-RGHFFHGIIPQNESLT
ABI33937_Echis_ocellatus                  LRYKVDLDLKTFHIVANANDTLSGSAVTIFYPTHLGIYPHIDD-RGHFFHGIIPQNESLT
ABI33938_Cerastes_cerastes                LRYKVDLDLKTFHIVSNANDSLSGSAVTIFYPNHLGVYPHIDD-RGHFFHGIIPQNESLT
ABI33941_Echis_pyramidum_leakeyi          LRYKVDLDLKTFHIVSNANDSLSGSAVTIFYPNHLGVYPHIDD-RGHFFHGIIPQNESLT
TR15727|c7_g3_i1_Potamotrygon_amandae     LRYDVDLDLSVFDISSNANDTLSGSVITIFYHNYLGKYPYYTK-ENVPVNGGIPQNSDLQ
TR10580|c4_g1_i1_Potamotrygon_falkneri    LRYDVDLDLSVFDISSNANDTLSGSVITIFYHNYLGKYPYYTK-ENVPVNGGIPQNSDLQ
XP_007896746_Callorhinchus_milii          LRYKVDLDLSIFDIASNSNETLSGSNITIFYHNQLGYYPYFTD-SKTPVNGGIPQNKSLQ
XP_006012182_Latimeria_chalumnae          LRYKVNLDLRVFDIVTNPNETLSGSNVTIFYHDRLGYYPYYSH-HKKPVNGGLPQNESLA
XP_009685663_Struthio_camelus_australis   LRYKVDLDLSVFDIASNTNETLSGSNVTIFYHTHLGYYPYYSD-NGDPVNGGVPQNESLI
XP_010213407_Tinamus_guttatus             LRYKVDLDLSIFDIASNTNETLSGSNVTIFYHTHLGYYPYYSD-NGDPVNGGVPQNESLI
XP_008943867_Merops_nubicus               LRYKVDLDLSVFDIASNTNETLSGSNVTIFYHTHLGYYPYYSD-NGDPVNGGVPQNESLI
XP_013054655_Anser_cygnoides_domesticus   LRYKVDLDLSIFDITSNTNETLSGSNVTIFYHTHLGYYPYYSD-NGDPVNGGVPQNESII
NP_001295181_Gallus_gallus                LRYKVDLDLSIFDIASNTNETLSGSNVTIFYHTHLGYYPYYSD-NGDPVNGGVPQNESLI
XP_006024588_Alligator_sinensis           LRYKVDLDLSVFDIASNTNETLSGSNVTIFYHTHLGHYPFYSD-NGYPVNGGVPQNESLI
XP_006268718_Alligator_mississippiensis   LRYKVDLDLSVFDIASNTNETLSGSNVTIFYHTHLGHYPFYSD-NGYPVNGGVPQNESLI
XP_005305767_Chrysemys_picta_bellii       LRYKVDLDLRVFDIASNTNETLSGSNVTIFYHTHLGHYPYYLD-NGVPINGGVPQNESLI
EMP32607_Chelonia_mydas                   LRYKVDLDLSVFDIASNTNETLSGSNVTIFYHTHLGHYPYYLD-TGVPVNGGVPQNESLI
XP_006122657_Pelodiscus_sinensis          LRYNVDLDLSVFDIASNINESLSGSNVTIFYHTHLGHYPYYLD-NGVPVNGGVPQNESLI
XP_006633469_Lepisosteus_oculatus         LKFKVDLDLSIFDIVANLNETLSGSNVTIFYHTHLGYYPFYSD-DGAPINGGLPQNQSLA
XP_012678206_Clupea_harengus              LRFKVDLDLSVFDIVANHNETLSGPNVTIFYHSHLGHYPYYTS-AGVPVNGGLPQNQSLT
NP_001074140_Danio_rerio_                 LRFKVDLDLSVFDIVANHNETLSGPNVTIFYHSHLGYYPYYTS-SETPINGGLPQNQSLS
XP_007246617_Astyanax_mexicanus           LRFKVDLDLSVFDIVANQNETLSGPNVTIFYHSHLGYYPYYTS-TGVPINGGLPQNQSLS
XP_005810535_Xiphophorus_maculatus        LRFKVDLDLSIFDIVANLNETLSGPNVTIFYHGHLGYYPYYSS-SGVAINGGLPQNQSIS
XP_003449275_Oreochromis_niloticus        LRFKVDLDLSIFDIVANLNETLSGPNVTIFYHSHLGYYPYYSN-SGIPINGGLPQNQSIS
XP_003967307_Takifugu_rubripes            LRFKVDLDLSVFDIVANLNETLSGPNVTIFYHSHLGYYPYYAV-SGEPINGGLPQNQSIS
ACI32917_Salmo_salar                      PRHGVRFPLEQFQIVASPNEGFVRQNLTIFYKDRLGLYPYYER-DGTAVNGGLPQAASLM
NP_001119986_Xenopus_tropicalis           PRFNVHLDLKLFDLNASPNEGFVDQNLTIFYKERLGMYPYYDE-HLAPVAGGLPQNASLR
                                           :  : . :  * .  .         : :**   :* :*             :**   :
```

```
EOB06026_Anas_platyrhynchos                 NHLKKAKEDIQFYIPSDEQSGLAVIDWENWRPLWIRNWGSKDIYRQESIDLVQQRDISLS
EMC77798_Columba_livia                      NHLKKAKEDIQFYIPSDEQFGLAVIDWENWRPVWIRNWGSKDIYRQESIELVQQRDLSLS
XP_002190553_Taeniopygia_guttata             NHLKKAREDIQFYIPSDEQLGLAVIDWENWRPVWIRNWGSKDIYRQESIELVQQRDLSIS
BAM66299_Pterois_antennata                  EHYNLAEDDIKYYMPSN-QPGLAVLDFEEWRPQWIRNWGSKDIYRRVSIETVKNKNASLS
BAM66300_Pterois_volitans                   EHYNLAEDDIKYYMPSN-QPGLAVLDFEEWRPQWIRNWGSKDIYRRVSIETVKNKNASLS
AAO74499_Synanceia_horrida                  QHHELAEDDIEYYIPFN-QLGLAVLDFEEWRPQWIRNWGSKDIYRQYSIETVLKKNSSLS
BAJ54082_Synanceia_verrucosa                EHHELAEDDIEYYIPFN-QLGLAVLDFEEWRPQWIRNWGSKDIYRQYSIETVLKKNSSLS
NP_001094250_Rattus_norvegicus              VHLEKAYQDINYYIPSENFSGLAVIDWEYWRPQWARNWNTKDIYRQKSRTLISDMKENIS
XP_001370718_Monodelphis_domestica          AHLEKAGQDINYYIPAEDFSGLAVIDWEYWRPQWARNWNTKDVYRQKSRKLISEVKVNMS
XP_001502439_Equus_caballus                 VHLEKADEDINYYIPAEDFSGLAVIDWEYWRPQWARNWNTKDVYRQKSRKLISDMQENVS
XP_002752104_Callithrix_jacchus             VHLEKVDQDINYYIPAEDFSGLAVIDWEYWRPQWARNWNAKDVYRQKSRKLISDRGKNVS
XP_002818436_Pongo_abelii                   VHLEKADQDINYYIPAEDFSGLAVIDWEYWRPQWARNWNAKDVYRQKSRKLISDMGKNVS
AAI04791_Homo_sapiens                       VHLEKADQDINYYIPAEDFSGLAVIDWEYWRPQWARNWNSKDVYRQKSRKLISDMGKNVS
XP_527872_Pan_troglodytes                   VHLEKADQDINYYIPAEDFSGLAVIDWEYWRPQWARNWNAKDVYRQKSRKLISDMGKNVS
XP_003217668_Anolis_carolinensis            DHLQKAQQDIKEVIEEVGFEGLAVVDWESWRPLWVRNWDSMEIYQNKSIDLVKEKHPDWP
ABI33944_Bitis_arietans                     KHLNKSKSDINRMIPLKTFHGLGVIDWENWRPQWDRNWGSKNVYRNRSIQFAKELHPELS
ABI33937_Echis_ocellatus                    KHLDKSKSDINRIIPLKTFHGLGVIDWENWRPQWDRNWGNKNVYRNRSIQFARDLHPELS
ABI33938_Cerastes_cerastes                  KHLNKSKSDINRIIPLKAFHGLGVIDWENWRPQWDRNWGSKNVYRNRSIQFARDLHPELS
ABI33941_Echis_pyramidum_leakeyi            KHLNKSKSDINRIIPLKAFHGLGVIDWENWRPQWDRNWGSKNVYRNRSIQFARDLHPELS
TR15727|c7_g3_i1_Potamotrygon_amandae       EHLNKTKSDIAKSIPYNDFSGLGVIDWEEWRPLWIRNWGDKDIYRQKSMELVKKLHPSWT
TR10580|c4_g1_i1_Potamotrygon_falkneri      EHLNKTKSDIAKSIPYNDFSGLGVIDWEEWRPLWIRNWGDKDIYRQKSMELVKKLHPSWT
XP_007896746_Callorhinchus_milii            DHLNKAKSDIEKSIPRKDFGGLGVIDWENWRPQWIRNWGHKNIYRKKSIGIERTLHPNWT
XP_006012182_Latimeria_chalumnae            KHLKKAESDIDRFLPFKNFHGLGVIDWENWRPQWIRNWGLKDIYRNKSKELVKKYHPHWS
XP_009685663_Struthio_camelus_australis     KHLNKAKSDIDYSIPMKKFQGLAVIDWENWRPQWDRNWGNKTIYRNKSLEMVRRRHPQWS
XP_010213407_Tinamus_guttatus               KHLNKAKSDIDYSIPMKKFQGLAVIDWENWRPQWDRNWGNKTIYRNKSLEMVRQRHPQWS
XP_008943867_Merops_nubicus                 KHLNKAKSDIDYCIPMKKFQGLAVIDWENWRPQWDRNWGNKSIYRNKSLEMVRRRHPQWS
XP_013054655_Anser_cygnoides_domesticus     KHLNKAKSDIDYCIPMKKFQGLAVIDWENWRPQWDRNWGSKSIYRNKSLEMVRRQHPQWS
NP_001295181_Gallus_gallus                  KHLNKAKSDIDYCIPMKKFHGLAVIDWENWRPQWDRNWGNKSIYRNKSLEMVRQRHPQWS
XP_006024588_Alligator_sinensis             KHLNKAKSDIDYSIPMKKFQGLAVIDWENWRPQWDRNWGNKTIYRNKSLELVRRRHPQWS
XP_006268718_Alligator_mississippiensis     KHLNKAKSDIDYSIPMKKFQGLAVIDWENWRPQWDRNWGNKTIYRNKSLELVRRRHPQWS
XP_005305767_Chrysemys_picta_bellii         KHLNKAKSDIDHSIPMKKFQGLAVIDWENWRPQWDRNWGNKTIYRNKSLELVRRHHPQWS
EMP32607_Chelonia_mydas                     KHLNKAKSDIDHSIPMKKFQGLAVIDWENWRPQWDRNWGNKTIYRNKSLELVRRCHPQWS
XP_006122657_Pelodiscus_sinensis            KHLNKAKSDIDHSIPMKKFQGLAVIDWENWRPQWDRNWGNKTIYRNKSFELVKRRHPQWS
XP_006633469_Lepisosteus_oculatus           KHLSKARSDIDKFIPFKDFKGLGVIDWENWRPQWVRNWGSKDIYRNKSKELIRRLHPQWS
XP_012678206_Clupea_harengus                KHLSKARADIDKLIPFKDFRGLGVIDWENWRPQWVRNWGSKDIYRNKSKELIRRIHPNWP
NP_001074140_Danio_rerio_                   KHLSKARADIDKLIPIKDFHGLGVIDWENWRPQWVRNWGSKDIYRNKSKELIRKLHPGWP
XP_007246617_Astyanax_mexicanus             KHLNKARADIDKLIPFKDFRGLAVIDWENWRPQWVRNWGSKDIYRNKSKELIRKAHPRWP
XP_005810535_Xiphophorus_maculatus          KHLNKARVDIDKLIPHKDFRGLGVIDWENWRPQWVRNWGSKDIYRKKSKEQIHKLHPHWP
XP_003449275_Oreochromis_niloticus          KHLGKARSDIDKLIPHKDFRGLGVIDWENWRPQWVRNWGSKDIYRKRSREQIRKLHPNWP
XP_003967307_Takifugu_rubripes              KHLTKARADIEKLIPQKDFRGLGVIDWENWRPQWVRNWGSKDIYRIKSKEQIRNLHPNWP
ACI32917_Salmo_salar                        QHYEKMPEGVHKYISEPNAKGLAVIDWEEWRPLWIRNWDVKDVYRNQSRQLVANKNLEWP
NP_001119986_Xenopus_tropicalis             AHLDKLPEGIQKYIRSRDKDGLAVIDWEEWRPIWVRNWQNKDVYRQNSRNLVSSRHPTWP
                                             *       :   :      **.*:*:* *** * ***    :*:  *

EOB06026_Anas_platyrhynchos                 EAEARTTAKMKFEAAAKSIMLESLKLGIAMKPKRLWGYYLYPDCYNYDYKQNPHNYTGTC
EMC77798_Columba_livia                      EAEARAIAKTEFEAAAKSIMLESLKLGIEMRPNRLWGYYLYPDCYNYDYNQNPHNYTGTC
XP_002190553_Taeniopygia_guttata            EAESRTIAKMEFEFAAKSFMLETLKLGIETKPNRLWGYYLYPDCYNYDYKQNPHNYTGAC
BAM66299_Pterois_antennata                  DNEAENQAKIEFERAARRYFVHSIRIGRRLRPNRLWGYYLFPDCYNYDFNKNMASFTGQC
BAM66300_Pterois_volitans                   DNEAENQAKIEFERAARRYFVHSIRIGRRLRPNRLWGYYLFPDCYNYDFNKNMASFTGQC
AAO74499_Synanceia_horrida                  KEEAAGQAKMAFERAAKKYFLRSIRIGKRLRPNRLWGYYLYPECYNYEYKKDMAGYTGEC
BAJ54082_Synanceia_verrucosa                KDEAAGQAKMAFERAAKKYFLRSIRIGKRLRSNRLWGYYLYPECYNYEYKKDMAGYTGEC
NP_001094250_Rattus_norvegicus              TADIEYLAKATFEKSAKAFMEETIKLGIKSRPKGFWGYYLYPDCHNYNFYA--TNYTGSC
XP_001370718_Monodelphis_domestica          ASEIEYLAKISFEESAKAFMKETIQLGIRSRPRGLWGYYLYPDCHNYNIYD--QNYTGSC
XP_001502439_Equus_caballus                 ATDIEYLAKATFEESAKAFMKETIELGIKSRPKGLWGYYLYPDCHNYNVYD--PNYTGSC
XP_002752104_Callithrix_jacchus             ATDIEYLAKVTFEESAKAFMKETIKRGIKSRPKGLWGYYLYPDCHNYNVYA--PNYTGSC
XP_002818436_Pongo_abelii                   ATDIEYLAKVTFEESAKAFMKETIKLGIKSRPKGLWGYYLYPDCHNYNVYA--PNYTGSC
AAI04791_Homo_sapiens                       ATDIEYLAKVTFEESAKAFMKETIKLGIKSRPKGLWGYYLYPDCHNYNVYA--PNYSGSC
XP_527872_Pan_troglodytes                   ATDIEYLAKVTFEESAKAFMKETIKLGIKSRPKGLWGYYLYPDCHNYNVYA--PNYTGSC
XP_003217668_Anolis_carolinensis            ADKVARTAQLEFEQSGRAFMEQTLALGKTLRPGGFWGFYGFPNCYNYDYKN--ASYTGDC
ABI33944_Bitis_arietans                     EDKIKRLAKKEYEKAAKSFMRDTLLLAEEMRPNGYWGYYLYPDCQNYDYKTKGDQYTGKC
ABI33937_Echis_ocellatus                    ENKIRRLAKAEYEKAAKSFMRDTLLLAEEMRPDGYWGYYLYPDCQNYDYKTKGDQYTGKC
ABI33938_Cerastes_cerastes                  EDKIRRLAKKEYEKAAKSFMRDTLLLAEEMRPDGYWGYYLYPDCQNYDYKTKGDQYTGKC
ABI33941_Echis_pyramidum_leakeyi            EDKIRRLAKKEYEKAAKSFMRDTLLLAEEMRPDGYWGYYLYPDCQNYDYKTKGDQYTGKC
TR15727|c7_g3_i1_Potamotrygon_amandae       DNEIKLEAQKEYETAALDLMNDTLESVEKLRPHGLWGFYLFPDCYNHHYKDQTKKYDGKC
TR10580|c4_g1_i1_Potamotrygon_falkneri      DNEIKLEAQKEYETAALDLMNDTLESVEKLRPHGLWGFYLFPDCYNHHYKDQTKKYDGKC
XP_007896746_Callorhinchus_milii            EKKIKSEAKKKFENAGRNFLNATLNLAEKTRPKGLWGFYLFPDCYNHNYKTHPNKYNGKC
XP_006012182_Latimeria_chalumnae            VKKIRKEARKQFEAAGKDFMNRTLLLAEQMRPNGLWGYYLFPDCYNYDYKKDPHKYTGKC
XP_009685663_Struthio_camelus_australis     EDKIRKVAKEEFENAGKSFMNNTILLAEHMRPNGLWGYYLYPDCYNYDYKEHPETYTGKC
XP_010213407_Tinamus_guttatus               EDKVRKVAKEEFENAGKSFMNNTILLAEHMRPNGLWGYYLYPDCYNYDYKEQPETYTGKC
XP_008943867_Merops_nubicus                 EDKIRKVAKEEFENAGRSFMNNTILLAEHMRPNGLWGYYLYPDCYNYDYKEHPRTYTGKC
XP_013054655_Anser_cygnoides_domesticus     EDKIRKVAKEEFENAGKSFMNNTILLAEHMRPNGLWGYYLYPDCYNYDYKEHPQTYTGKC
NP_001295181_Gallus_gallus                  EDKIRKVAKEEFENAGKSFMNNTILLAEHMRPNGLWGYYLYPDCYNYDYKEHWQTYTGKC
XP_006024588_Alligator_sinensis             EDKLRKVAKEEFENAGKSFMNNTILLAEHMRPNGLWGYYLYPDCYNYDYKEHPQAYTGKC
XP_006268718_Alligator_mississippiensis     EDKLRKVAKEEFENAGKSFMNNTILLAEHMRPNGLWGYYLYPDCYNYDYKEHPQAYTGKC
XP_005305767_Chrysemys_picta_bellii         EDKIRKVAKEEFENAGKSFMNNTILLAEHMRPNGLWGYYLYPDCYNYDYKEHPKVYTGRC
EMP32607_Chelonia_mydas                     EDKIRKVAKEEFENAGKSFMNNTILLAEHMRPNGLWGYYLYPDCYNYDYKEHPKAYTGRC
XP_006122657_Pelodiscus_sinensis            EDKIRKVAKEEFENAGKNFMNNTILLARHMRPNGLWGYYLYPDCYNYDYKDQPKAYTGKC
XP_006633469_Lepisosteus_oculatus           DRKVEKEAKEEFEMAGLNFMNTTLLLAENRRPNGIWGFYLYPDCYNYGYKQHPQKYTGEC
XP_012678206_Clupea_harengus                QSKVEHEAKELFERAGQAFMNTTLRLAQSRRPHGLWGFYLFPDCYNYGYKQHPQRYTGAC
NP_001074140_Danio_rerio_                   QSKVENEAKEVFERAGQVFMNSTLLLAESRRPHGLWGFYLFPDCYNYGYKQHPFRYTGEC
XP_007246617_Astyanax_mexicanus             QSKVENEAKETFERAGQAFMNFTLQLAKSRRPHGLWGFYLFPDCYNYGYKQHPHRYTGEC
XP_005810535_Xiphophorus_maculatus          ESKVEKEAKDRFEKSGQAFMNLTLALAEARRPDGLWGFYLFPDCYNYGYKQHPQCYTGEC
XP_003449275_Oreochromis_niloticus          ESKVEKEAKEGFERAGQAFMNLTLALAQGRRPGGLWGFYLFPDCYNYGYKQHPQHYTGEC
XP_003967307_Takifugu_rubripes              DSKIEKEAKESFERAGLAFMNLTLALAEGRRPDGLWGFYLFPDCYNYGYKQHPQRYTGEC
ACI32917_Salmo_salar                        LERVGKVAQQEFELSARKFMLETLRLAKSLRPNQLWGFYLFPDCYNHDYRSGLENYTGRC
NP_001119986_Xenopus_tropicalis             REKVDKEALYEFENAAREFMTETLRHAKNYRPRQLWGFYLFPDCYNHDYVKNRDSYTGQC
                                                   *   :* :.   :  ::      :    **:* :*:* *:        : * *
```

```
EOB06026_Anas_platyrhynchos              LDIEIARNNELNWLWEKSTALYPSVYLETALRSSRNAQLFVRNRVQEAIRVSYVSNSTYP
EMC77798_Columba_livia                   LDIEIERNNELKWLWEESTALYPSVYLETALRSSRNAQLFVRNRVQEAMRISYVSNSTHP
XP_002190553_Taeniopygia_guttata         LDIEIERNNELNWLWEKSTALYPSIYLETALKSSRNAQLFVRNRVQEAIRTSYVANSSHP
BAM66299_Pterois_antennata               PALEKKRNDELMWLWRESTALFPSIYLELVIRDSQQARQYVRHRIQEAMRVSMLPNSNYS
BAM66300_Pterois_volitans                PALEKKRNDELMWLWRESTALFPSIYLELVIRDSQQARQYVRHRIQEAMRVSMLPNSNYS
AAO74499_Synanceia_horrida               PAIEKDRNNELLWLWRESTALFPSIYLELLLRDTQQARQYVRHRIQESIRVSKLPNSAYS
BAJ54082_Synanceia_verrucosa             PAIEKGRNDELLWLWRESTALFPSIYLELLLRDTEQARQYVRHRIQESIRVSKLPNSAYS
NP_001094250_Rattus_norvegicus           PEEEVLRNNDLSWLWNSSTALYPAVSIRKSFADSENTLHFSQFRVRESLRISTMTSHDYA
XP_001370718_Monodelphis_domestica       PEDEVLRNNELSWLWNSSAALYPSIGVKKSFGNSGNILRFSQFRVNESMRISTMTSHDYA
XP_001502439_Equus_caballus              PEEEVLRNNELSWLWNSSAALYPSIGVRKSLGDSENILRFSQFRVHESMRISTMTSHDYA
XP_002752104_Callithrix_jacchus          PEEEILRNNELCWLWNSSAALYPSVGIRKSLGGNENILRFSQFRVHESMRISTMTSYDYA
XP_002818436_Pongo_abelii                PEDEVLRNNELSWLWNSSAALYPSIGVWKSLGDSENILRFSKFRVHESMRISTMTSHDYA
AAI04791_Homo_sapiens                    PEDEVLRNNELSWLWNSSAALYPSIGVWKSLGDSENILRFSKFRVHESMRISTMTSHDYA
XP_527872_Pan_troglodytes                PEDEVLRNNELSWLWNSSAALYPSIGVWKSLGDSENILRFSKFRVHESMRISTMTSHDYA
XP_003217668_Anolis_carolinensis         PETEKQRNEELQWLWNQSRALYPSIYLPKDLEFLNKTLKFVRHRIQEAFRMANQS-GDGS
ABI33944_Bitis_arietans                  PDIEMSRNDQLLWLWRDSTALFPNVYLEIILRSSDNALKFVHHRLKESMRIASMAREDYA
ABI33937_Echis_ocellatus                 PDIEMSRNDQLLWLWRESTALFPNVYLEIILRSSDNALKFVHHRLKESMRIASMAREDYA
ABI33938_Cerastes_cerastes               PEIEMSRNDQLLWLWRDSTALFPNVYLEIILRSSDNALKFVHHRLKEAMRIASMAREDYA
ABI33941_Echis_pyramidum_leakeyi         PEIEMSRNDQLLWLWRDSTALFPNVYLEIILRSSDNALKFVHHRLKEAMRIASMAREDYA
TR15727|c7_g3_i1_Potamotrygon_amandae    PPIEYKRNDKLLWLWKKSGALYPSIYLGLDLKSSPKARLFVHYRLKEAIRLASMAHKDYA
TR10580|c4_g1_i1_Potamotrygon_falkneri   PPIEYKRNDKLLWLWKKSGALYPSIYLGLDLKSSPKARLFVHYRLKEAIRLASMAHKDYA
XP_007896746_Callorhinchus_milii         PQIEHKRNDKLLWLWRESTALFPSIYLERQLKSSPKALKFVHYRVKEAMRIASMARSDYD
XP_006012182_Latimeria_chalumnae         PDIEYIRNDRLLWLWEESTALYPSIYLEKKLKSTANALKFVHYRVKEAVRVASIAKKDYA
XP_009685663_Struthio_camelus_australis  PAIESSRNDLLLWLWKESTALYPSIYLDYMLKSSPNALKFVHYRVKEAIRIASIARKDYL
XP_010213407_Tinamus_guttatus            PDIESSRNDLLFWLWKESTALYPSIYLDYILKSSPNALKFVHYRVKEAIRIASIARKDYV
XP_008943867_Merops_nubicus              PPIESSRNDLLLWLWKESTALYPSIYLDYVLKSSPNALKFVHYRVKEAIRIASIARKDYV
XP_013054655_Anser_cygnoides_domesticus  PAIESSRNDLLLWLWKESTALYPSIYLDYVLKSSPNALKFVHYRVKEAIRIASIARKDYV
NP_001295181_Gallus_gallus               PAIESSRNDLLLWLWKESTALYPSIYLDYILKSSPNAQKFVHYRVKEAIRVASIARKDYV
XP_006024588_Alligator_sinensis          PDIESARNDLLIWLWKESTALYPSIYLDYILKSSSNALKFVHYRVKEAIRIASIARKDYI
XP_006268718_Alligator_mississippiensis  PDIESARNDLLIWLWKESTALYPSIYLDYILKSSSNALKFVHYRVKEAIRIASIARKDYI
XP_005305767_Chrysemys_picta_bellii      PDIESSRNDLLLWLWKESTALYPSIYLDYILKSSPNALKFVHYRVKEAIRIASVARKDYV
EMP32607_Chelonia_mydas                  PDIESSRNDLLLWLWKESTALYPSIYLDYILKSSPNALKFVHYRVKEAIRIASVARKDYV
XP_006122657_Pelodiscus_sinensis         PAIESSRNDLLLWLWKESTALYPSIYLDYILKSSPNALKFVHYRVKEAIRIASIARKDYV
XP_006633469_Lepisosteus_oculatus        PNIEHVRNDHLMWLWKESTALYPSIYLDYELKSTSNTVKFVHYRVKEAMRIASIARTDHM
XP_012678206_Clupea_harengus             PNVEHVRNDHLMWLWRESTALYPSIYLDYELKSSPNTVKFVHYRVKEAMRIASIVRNDFT
NP_001074140_Danio_rerio_                PNIEHVRNDHLMWLWKESTALYPSIYLDCELKSSTNTVKFVHYRVKEAMRIASIARNDYT
XP_007246617_Astyanax_mexicanus          PNVEHVRNDHLMWLWKESTALYPSIYLDYELKSSPNTVKFVHYRVKEAMRIASIARNDFT
XP_005810535_Xiphophorus_maculatus       PNVEHVRNDHLMWLWKKSTALYPSIYLDFELKSSSNTIKFVHYRVKEAMRIATIAHTDYT
XP_003449275_Oreochromis_niloticus       PNVEHVRNDHLIWLWKESSALYPSIYLDYELKSSSNTVKFVHYRVKEAMRIASIARTDFT
XP_003967307_Takifugu_rubripes           PNVEHVRNDHLMWLWKESTALYPSIYLDYELKSSPNTVKFVHYRVKEALRIASIARTDFT
ACI32917_Salmo_salar                     PDVEVARNDQLTWLWTESTALFPSVYMSTVLRSTMFGCQFVRNRVKEGMRLASA-GDGLA
NP_001119986_Xenopus_tropicalis          PDVEISRNDQLSWLWEESTALYPSIYLDQILASSENGRKFVRSRVREAMRISYRHHKDYS
                                            *  **: * *** .* **:* : :   :        : : *:.*..* :

EOB06026_Anas_platyrhynchos              LPVFVYTRPVFTDVYEEYLSQDDLVNTIGESAALGASGVVIWGDLNLTQSKNTCKTLDNY
EMC77798_Columba_livia                   LPVIVYTRPVFTDVYEEYLSQDDLVNTIGESAALGASGIVIWGDMNLTQNKNTCRTLDNY
XP_002190553_Taeniopygia_guttata         LPVFVYTRPVFTDVYEEYLSEDDLVNTIGESAALGASGIVIWGDMNLTQNKNTCRTLDNY
BAM66299_Pterois_antennata               IPIHAYIRPLYKDTTDIYMSEFDLVNTIGEAAALGAAGVISWGDMNVVKTEDSCFDAKSH
BAM66300_Pterois_volitans                IPIHAYIRPLYKDTTDIYMSEFDLVNTIGEAAALGAAGVISWGDMNVVKTEDSCFDAKSH
AAO74499_Synanceia_horrida               IPIHAYVRPVYKDSTDNYMSEFDLVNTIGEAAALGAASVVCWGDMSVVATEDSCFDARRH
BAJ54082_Synanceia_verrucosa             IPIHAYIRPVYKDSTDDYMSEFDLVNTIGEAAALGAASVVCWGDMNVVATEDSCFDARRH
NP_001094250_Rattus_norvegicus           LPVFVYTRLGYKEEPLLFLSKQDLISTIGESAALGAAGIVVWGDMNLTSSAENCTKVNRF
XP_001370718_Monodelphis_domestica       LPVFVYTRLGYRDEPLYFLSKQDLISTIGESAALGAAGIVIWGDMNLTSSEGNCTKVKQF
XP_001502439_Equus_caballus              LPVFVYTRLGYRDEPLFFLSKQDLISTIGESAALGAAGFVIWGDMNLTSSEGNCTKVKQF
XP_002752104_Callithrix_jacchus          LPVFVYTRLGYRDEPLVFLSKQDLVSTIGESAALGAAGIVIWGDMNLTSSKANCTKVKQF
XP_002818436_Pongo_abelii                LPVFVYTRLGYRDEPLFFLSKQDLVSTIGESAALGAAGIVIWGDMNLTSSKANCTKVKQF
AAI04791_Homo_sapiens                    LPVFVYTRLGYRDEPLFFLSKQDLVSTIGESAALGAAGIVIWGDMNLTSSKANCTKVKQF
XP_527872_Pan_troglodytes                LPVFVYTRLGYRDEPLFFLSKQDLVSTIGESAALGAAGIVIWGDMNLTSSKANCTKVKQF
XP_003217668_Anolis_carolinensis         IPVLPYASLAYEF-TQIFLTQEDLAHTIGESASQGAAGVILWGSSNYSYSQEACLGVKKY
ABI33944_Bitis_arietans                  LPVFVYARPFYAY-TFEPLTQEDLVTTVGETAAMGAAGIVFWGSMQYASTVDSCQKVKTY
ABI33937_Echis_ocellatus                 LPVFVYARPFYAY-TFEPLTQEDLVTTVGETAAMGAAGIVFWGSMQYASTVDSCQKVKKY
ABI33938_Cerastes_cerastes               LPVFAYARPFYAY-TFEPLTQEDLVTTVGETAAMGAAGIVFWGSMQYASTVDSCQKVKKY
ABI33941_Echis_pyramidum_leakeyi         LPVFAYARPFYAY-TFEPLTQEDLVTTVGETAAMGAAGIVFWGSMQYASTVDSCQKVKKY
TR15727|c7_g3_i1_Potamotrygon_amandae    IPVFAYGRPYYTY-ELIPLTEIDLIHTIGESAAMGTSGVILWGGMDYARSKTHCLAVKEY
TR10580|c4_g1_i1_Potamotrygon_falkneri   IPVFAYGRPYYTY-ELIPLTEIDLIHTIGESAAMGTSGVILWGGMDYARSKTHCLAVKEY
XP_007896746_Callorhinchus_milii         LPVFVYARPFYAY-RFEVLTKIDLVHTIGESAAMGAAGIVLWGNIDYARSKNHCLKVKKY
XP_006012182_Latimeria_chalumnae         LPIFVYSRPFYTH-SIQALTEEDLISTIGQSAAMGAAGVVLWGSMQYARSKKNCLLVKEN
XP_009685663_Struthio_camelus_australis  LPVFVYSRPFYAY-TFHILTETDLVNTIGESAALGAAGVVLWGSIQYASSKESCSTVKRY
XP_010213407_Tinamus_guttatus            LPVFVYSRPFYAY-TFRVLTEIDLVHTIGESAALGAAGVVLWGSIQYASSKESCSVVKRY
XP_008943867_Merops_nubicus              LPVFVYSRPFYAY-TFHVLTEVDLVNTIGESAALGAAGVVLWGSMQYASSKESCSTVKRY
XP_013054655_Anser_cygnoides_domesticus  LPVFVYSRPFYAY-TFHVLTETDLVNTIGESAALGAAGVVLWGSMQYASSKESCSTVKRY
NP_001295181_Gallus_gallus               LPVFVYSRPFYAY-TFHVLTEIDLVNTIGESAALGAAGVVLWGSMQYASSKESCSTVKQY
XP_006024588_Alligator_sinensis          LPVFVYSRPFYAY-TFYVLTEIDLVNTIGESAALGAAGVVLWGSMQYASSQESCSTVKRY
XP_006268718_Alligator_mississippiensis  LPVFVYSRPFYAY-TFYVLTEIDLVNTIGESAALGAAGVVLWGSMQYASSQESCLTVKRY
XP_005305767_Chrysemys_picta_bellii      LPVFVYSRPFYAY-TLHVLTETDLVNTIGESAALGAAGVVLWGSMQYASSKESCSVVKKY
EMP32607_Chelonia_mydas                  LPVFVYSRPFYAY-TLHVLTETDLVNTIGESAALGAAGVVLWGSMQYASSKESCSVVKKY
XP_006122657_Pelodiscus_sinensis         LPVFVYSRPFYAY-TLHVLTKTDLVNTIGESAALGAAGVVLWGSMQYASSEENCSIVKKY
XP_006633469_Lepisosteus_oculatus        LPVYVYSRPFYAH-TFHVLSEIDLVHTIGESAALGAAGVVLWGSSEYARSRKNCLTVKKY
XP_012678206_Clupea_harengus             LPVFVYSRPFYAY-TFVVLTETDLVHTIGESAALGAAGVVLWGSSEYARSERNCLTVKKY
NP_001074140_Danio_rerio_                LPVFVYSRPFYAY-TFVVLSESDLVHTIGESAALGAAGVVLWGSSEYARSQRNCLTVKKY
XP_007246617_Astyanax_mexicanus          LPVFVYSRPFYAY-TFTVLSESDLVHTIGESAALGADGVVLWGSSEYARSQRNCLTVKKY
XP_005810535_Xiphophorus_maculatus       LPVFVYSRPFYAY-TFVVLSESDLVHTIGESAALGASGVVLWGSSEYARSKRNCLTVKKY
XP_003449275_Oreochromis_niloticus       LPVFVYSRPFYAY-TFVVLSESDLIHTIGESAALGASGVVLWGSSEYARSQRNCLTVKKY
XP_003967307_Takifugu_rubripes           LPVFVYSRPFYAY-TFVILSESDLVHTIGESAALGASGIILWGSSEYARSQKNCLTVKKF
ACI32917_Salmo_salar                     RPVFVYTRPTYAN-ELTPLTETDLVSTIGESVALGASGVIIWGDHTYASSNASCSSLSEY
NP_001119986_Xenopus_tropicalis          LPVFVYTRPTYIR-KLDFLSQMDLISTIGESAAQGAAGVIFWGDAEYTKSKETCQMIKKY
                                          *:  *    :       :::  **  *:*::.: *: ..: **      .   *
```

```
EOB06026_Anas_platyrhynchos                  LKRTLTPYLINVTMAARICSQVLCQDSGACARKKWNSSDYLHLNPKNIVIQMT----KDG
EMC77798_Columba_livia                       LRRTLTPYLINVTMAARICSQVLCQDSGACARKKWNSSDYLHLNPENIVIQMT----KDG
XP_002190553_Taeniopygia_guttata             LRRTLTPYLINVTMAARICSQVLCQDSGACARKKWNSSDYLHLNPENIAIQMT----KDG
BAM66299_Pterois_antennata                   LDKVMNPYILNVTTATQLCSEALCQGLGRCVRKRWDDDVFLHLDPRRYRIERKH---RGG
BAM66300_Pterois_volitans                    LDKVMNPYILNVTTATQLCSEALCQGLGRCVRKRWDDDVFLHLDPRRYRIERKH---RGG
AAO74499_Synanceia_horrida                   LEKVMNPYIMNVSTATQLCSKALCQDQGRCVRKHWDDDVFLHLDPRRYRIEQQR---GGG
BAJ54082_Synanceia_verrucosa                 LEKVMNPYIMNVSTAAQLCSKALCQDRGRCVRKHWDDDVFLHLDPRRYRIERQS---RGG
NP_001094250_Rattus_norvegicus               VNSDFGSYVINVTTAAEVCSRHLCKNNGRCARKTWKAAHYLHLNPASYHIEAS----ADR
XP_001370718_Monodelphis_domestica           VTSDLGSYIVNVTKAAEVCSRRLCRNNGRCVRKIWKAADYLHLNPDNCQIEVS----DDG
XP_001502439_Equus_caballus                  VSSVLGRYIVNVTRAAEACSLHLCRNNGRCLRKVWKAPDYLHLNPASYYIEAS----EDG
XP_002752104_Callithrix_jacchus              VSSDLGSYIVNVTGAAEVCSFHLCRNNGRCIRKMWNAPSYLHLNPASYHIEAS----EDG
XP_002818436_Pongo_abelii                    VSSDLGSYIANVTRAAEVCSLHLCRNNGRCIRKMWNAPSYLHLNPTSYHIEAA----EDG
AAI04791_Homo_sapiens                        VSSDLGSYIANVTRAAEVCSLHLCRNNGRCIRKMWNAPSYLHLNPASYHIEAS----EDG
XP_527872_Pan_troglodytes                    VSSDLGSYIANVTRAAEVCSLHLCRNNGRCIRKMWNAPSYLHLNPASYHIEAS----EDG
XP_003217668_Anolis_carolinensis             VDTLLGPYIVNVTSSAKLCSQVVCSKNGRCVRQDNHPEAFLHLSPDHYTITKHP---KEP
ABI33944_Bitis_arietans                      MNGPLGRYIVNVTTAAKICSHALCRKNGRCVRKHSDSNAFLHLFPESFRIMVHA-NATEK
ABI33937_Echis_ocellatus                     MNGPLGRYIINVTTAAKICSHALCRKNGRCVRKHSDSNAFLHLFPESFRIMVHA-NATEK
ABI33938_Cerastes_cerastes                   MNGPLGRYIVNVTTAAKICSRVLCRKNGRCVRKHSDSNAFLHLFPESFRIMVYA-NATEK
ABI33941_Echis_pyramidum_leakeyi             MNGPLGRYIVNVTTAAKICSRVFCRKNGRCVRKHSDSNAFLHLFPESFRIMVYA-NATEK
TR15727|c7_g3_i1_Potamotrygon_amandae        IDGLLGHYIVNVTSATKLCSKHLCKTHGRCVRKNIDSKSYLHLDPRSFKITTNS-KGVHP
TR10580|c4_g1_i1_Potamotrygon_falkneri       IDGLLGHYIVNVTSATKLCSKHLCKTHGRCVRKNIDSKSYLHLDPRSFKITTNS-KGVHP
XP_007896746_Callorhinchus_milii             IDGPLGHYVVNVTTATKLCSKTLCENHGRCVRKISDSRVFLHLSPSTFEIRTNA-DGVHP
XP_006012182_Latimeria_chalumnae             IDGPLGHYVINVTSAAELCSKVLCSKNGKCIRQSLDSGTYLHLNPSSFTIKTNF-ATRGP
XP_009685663_Struthio_camelus_australis      IDGPLGHYVINVTSAAKLCSKVLCKKNGRCIRKNSDSAAYLHLSPTNFKIQVRH-SAMGP
XP_010213407_Tinamus_guttatus                IDGPLGHYVINVTSAAKLCSKVLCKKNGRCIRKNSDSSAYLHLSPQNFKIQVHH-SERGP
XP_008943867_Merops_nubicus                  VDGPLGHYVINVTSAAKLCSKVLCKKNGRCIRKNSDSSAYLHLSPTNFKIQTRH-SERGP
XP_013054655_Anser_cygnoides_domesticus      IDGPLGHYVINVTSAAKLCSKVLCKKNGRCIRKNSDSSAYLHLPPTNFKIRVRH-SGRGP
NP_001295181_Gallus_gallus                   IDGPLGHYVINVTSAAKLCSKVLCKKNGRCIRKNSDSSAYLHLPPSDFKIRARR-SGRGP
XP_006024588_Alligator_sinensis              IDGPLGHYVINVTAAAKLCSKVLCKKNGRCIRKNSESSAYLHLSSNSFKIRVHY-SEKGP
XP_006268718_Alligator_mississippiensis      IDGPLGHYVINVTAAAKLCSKVLCKKNGRCIRKNSESSAYLHLSSNSFKIRVHY-SEKGP
XP_005305767_Chrysemys_picta_bellii          IDGPLGHYVINVTSAAKLCSKVLCKKNGRCIRKIGDSSAYLHLSPNSFEIGVHH-SEKGP
EMP32607_Chelonia_mydas                      IDGPLGHYVINVTSAAKLCSKVLCKKNGRCIRKISDSSVYLHLSPNSFEIRVHH-SEKGS
XP_006122657_Pelodiscus_sinensis             INGPLGHYIINVTSAAKLCSKVLCKKNGRCIRKISGSSSYLHLSPKSFEIGVHH-SEKGP
XP_006633469_Lepisosteus_oculatus            IDGPLGHYVINVTSAAKLCSKALCKKNGKCVRKSLDNGAFLHLNPRYFKIRSSS-SSQGP
XP_012678206_Clupea_harengus                 IDGPLGHYVINVTSAAKLCSKALCKKNGKCVRKSLDSGAYLHLNPRFFHIHRNP-GPQGP
NP_001074140_Danio_rerio_                    IDGPLGHYVINVTSAAKLCSKALCKKNGKCVRKSLDSGAYLHLNPRFFNIRLNQ-GIRGP
XP_007246617_Astyanax_mexicanus              IDGPLGHYVINVTSAAKLCSKALCKKNGKCVRKSMDSKAYLHLNPRFFNIHLNR-AARGP
XP_005810535_Xiphophorus_maculatus           IDGPLGHYVINVTAAAKLCSKALCKKNGRCVRKSLDSRYYLHLNPRYFHIHHNP-APGGP
XP_003449275_Oreochromis_niloticus           INGPLGHYVINVTSAAKLCSKALCKKNGRCIRKSLDSDTYLHLNSRFFHIHHNS-APSGP
XP_003967307_Takifugu_rubripes               IDGPLGHYVINVTSATKLCSKALCKKNGRCIRKSLDSGAYLHLNPHFFHIRHDP-TLKGR
ACI32917_Salmo_salar                         LRGPLGRYLFNVSTAAELCSQKLCSFHGRCLRKHSDTDAYLHLSPLTHTIS-----SQGG
NP_001119986_Xenopus_tropicalis              LDEDLGHYIVNVTTAAELCSQSLCNGNGRCLRQENNTDAFLHLNPANFQIVSAPKDFQGP
                                             :   :  *: **: ::. **  .*   * * *:       :***     *

EOB06026_Anas_platyrhynchos                  KYTLQGQPAFQDLQTFIEKFDCHCYAGHT---CEPRVNINDIQYLRACISEDICIQISSN
EMC77798_Columba_livia                       KYTLQGQPTFQDLQTFIEKFDCHCYAGHS---CEPRADINDIHYLHICISEDICIQISSN
XP_002190553_Taeniopygia_guttata             KYTLQGQPSYQDLQTFIEKFDCHCYAGHS---CEPRADINDIHYLHACISEDICIQISSN
BAM66299_Pterois_antennata                   PLTVSGGLSQDDVNWFDRSFDCMCYTEQP---CRSVMIFNVINKTV--------------
BAM66300_Pterois_volitans                    PLTVSGGLSQDDVNWFDRRFDCMCYTEQP---CRSVMIFNVINKTV--------------
AAO74499_Synanceia_horrida                   PLTVTGDLSQDDVNWFDRNFDCMCYSEKP---CRSALTFNVINKAV--INKA--------
BAJ54082_Synanceia_verrucosa                 PLTVIGDLSQDDVNWFDRNFDCMCYSEKP---CRSTLTFNVINKAV--ISKA--------
NP_001094250_Rattus_norvegicus               EFVVKGRASDADLAAMAENFLCHCYEGYEGADCRDMTEASGPSGV-SVSS--------SS
XP_001370718_Monodelphis_domestica           DFAVKGEPSDSDLKVMAERFSCHCYQGYGGKDCREMKTSGHHSGV-SPSS--------GS
XP_001502439_Equus_caballus                  EFIVEGKASDTDLALMAEKFSCQCYQGYEGADCREMKTADGCSGL-PSFS--------GS
XP_002752104_Callithrix_jacchus              EFTVKGKASDTDLAVMADKFSCHCYQGYEGADCRQMKMADGCSGV-SPSP--------GS
XP_002818436_Pongo_abelii                    EFTVKGKASDTDLAVMADTFSCHCYQGYEGADCRETKTADGCSGV-SPSP--------GS
AAI04791_Homo_sapiens                        EFTVKGKASDTDLAVMADTFSCHCYQGYEGADCREIKTADGCSGV-SPSP--------GS
XP_527872_Pan_troglodytes                    EFTVKGKASDTDLAVMADTFSCHCYQGYEGADCREIKTADGCSGV-SPSP--------GS
XP_003217668_Anolis_carolinensis             GFILTGYLSKEDLVKMAEEFKCRCYSGWEGLRWIRLKLLAPFCTK------TVPFEYPWH
ABI33944_Bitis_arietans                      KAIVKGKLELKDLIYLRKNFMCQCYQGWKGLYCEEYSIKDIRKI----------------
ABI33937_Echis_ocellatus                     KVIVKGKLELENLIYLRENFMCQCYQGWQGLYCEEYSIKDIRKI----------------
ABI33938_Cerastes_cerastes                   KVIVKGKLELENLIYLRENFMCQCYQGWKGLYCEEYSIKDIRKI----------------
ABI33941_Echis_pyramidum_leakeyi             KVIVKGKLELENLIYLRENFMCQCYQGWKGLYCEEYSIKDIRKI----------------
TR15727|c7_g3_i1_Potamotrygon_amandae        RFSVKGKINQRVLKNMNEMFTCQCYEGWVGISCEIPAVDIFPVKK-D-V-PEVK--SERG
TR10580|c4_g1_i1_Potamotrygon_falkneri       RFSVKGKINQRVLKNMNEMFTCQCYEGWVGISCEIPAVDIFPVKK-D-V-PEVK--SERG
XP_007896746_Callorhinchus_milii             RFYVQGKLQSEDMKALKQSFACQCYQGWTGIACEVPLPEDPSAVK-SDISL----SEKAS
XP_006012182_Latimeria_chalumnae             KFFVTGEHTDEDVKAMKQKFMCQCYQGWVGMFCEQPDALATARLV-Q---------FPSG
XP_009685663_Struthio_camelus_australis      RFQVTGKLSLKNVEAMRQRFMCQCYQGWTGIFCELPDQRLMEHRV--------HFVFDRS
XP_010213407_Tinamus_guttatus                RFQVTGKPSLKNIKAMRQRFMCQCYQGWTGIFCELPDQRLMEHWV--------HFVFSRS
XP_008943867_Merops_nubicus                  RFQVTGEPSLDSIEAMRQKFMCQCYQGWTGIFCELPDQRLMERWV--------RVVFSRS
XP_013054655_Anser_cygnoides_domesticus      RLQVTGKPSLENIEAMRQRFMCQCYQGWTGIFCELPDQRLKEHWA--------HIVLSRS
NP_001295181_Gallus_gallus                   RLQVTGELSLENIEAMRQRFMCQCYQGWTGIFCELPDQSVMEHCV--------HIVFSRS
XP_006024588_Alligator_sinensis              RFVVTGKPGPEDIEAMKQRFMCQCYQGWTGIFCELPNQKLLGHLA---------------
XP_006268718_Alligator_mississippiensis      RFVVTGKPGPEDIEAMKQRFMCQCYQGWTGIFCELPNQKLLGHLA---------------
XP_005305767_Chrysemys_picta_bellii          RFFVTGKLRPEDMKAMKQSFTCQCYQGWTGIFCELPDRSLLEQWA--------HTLFNRS
EMP32607_Chelonia_mydas                      RFFVTGKPRPEDIKAMKQSFTCQCYQGWTGIFCELPDRSVLEQWA--------HTLFNRS
XP_006122657_Pelodiscus_sinensis             RFFVTGKPRPKDIQAMKQSFTCQCYQGWTGIFCELPDQSLFEQWV--------HTLFNRA
XP_006633469_Lepisosteus_oculatus            RFFVSGHLSNEDIFVMKQKFTCQCYHGWIGIYCEMPESSILF--------------VEYR
XP_012678206_Clupea_harengus                 RFTVRGHLNNHDILDMKHKFTCQCYQGWTGIYCEMPQTT-GLQSG-P------------S
NP_001074140_Danio_rerio_                    CFYVSGHLNNLDILDMKQKFTCQCYQGWTGIYCEMPQMAEPLPPH-P------------R
XP_007246617_Astyanax_mexicanus              RFHVSGHLNYHDILDMKHKFTCQCYQGWTGIYCEIPQLIHPLPPR-S------------N
XP_005810535_Xiphophorus_maculatus           RFEVRGHLNSHDILDMRHKFTCQCYQGWTGVYCEIPQTQPP------PLSPPVLITHPWG
XP_003449275_Oreochromis_niloticus           RFHISGHLNNHDILDMKQKFTCQCYQGWTGVYCEVPQTMPPHPLP-PPLQPTVPVLRPQG
XP_003967307_Takifugu_rubripes               RFHVSGHLNHDDILAMKQKFTCQCYKGWTGVYCEMPQAPP-CPPG-RLLHPAIHQPQPSQ
ACI32917_Salmo_salar                         RLKVTGQLGQGELAGYRQHFHCQCYSGYKGEGCAQRELGKSGAAP-V-------------
NP_001119986_Xenopus_tropicalis              SLRAEGKLSAGDIATLRSQFRCQCYVDWYGDSCGIQRSTNGGAVA-T-------------
                                                  *      :      * * **
```

```
EOB06026_Anas_platyrhynchos                 --SLSNIEASDEKILSNRTV------FSLTSQSKVT------------------------
EMC77798_Columba_livia                      --SVSNIEASEEKIPSNGTI------FSFTSPSKVTLSTHP-------------------
XP_002190553_Taeniopygia_guttata            --SLSNIEASEEKILSNRTA------FSFTSESKATLSTHPEVEDFQSTFGNNILNITTA
BAM66299_Pterois_antennata                  --------------------------FTS--KNGGARGPRPLLLVMK-LIC--------L
BAM66300_Pterois_volitans                   --------------------------FTS--KNGGARGPRPLLLVMK-LIC--------L
AAO74499_Synanceia_horrida                  --------------PPACDG---------------TARSDVHCDV---NIN--------F
BAJ54082_Synanceia_verrucosa                --------------VINRAV------FTS--RSRGAERPRPLLLVMV-LLG--------L
NP_001094250_Rattus_norvegicus              VITLCLIA-----LAGHQ-S---------------------------------I---Q-L
XP_001370718_Monodelphis_domestica          RIGQCLLV-----LVYYL-N---------------------------------L---Q-C
XP_001502439_Equus_caballus                 LITLCPLF-----LAGYQ-S---------------------------------I---Q-L
XP_002752104_Callithrix_jacchus             LITLCLLL-----LASYQ-S---------------------------------I---Q-L
XP_002818436_Pongo_abelii                   LMTLCLLL-----LASYR-S---------------------------------I---Q-L
AAI04791_Homo_sapiens                       LMTLCLLL-----LASYR-S---------------------------------I---Q-L
XP_527872_Pan_troglodytes                   LMTLCLLL-----LASY-------------------------------------------
XP_003217668_Anolis_carolinensis            V----QLQ----KFASYKKMFPVSEKVCLSSPSRGRLEAS-ICCMFS-PLCQAL---G-Q
ABI33944_Bitis_arietans                     ------------------------------------------------------------
ABI33937_Echis_ocellatus                    ------------------------------------------------------------
ABI33938_Cerastes_cerastes                  ------------------------------------------------------------
ABI33941_Echis_pyramidum_leakeyi            ------------------------------------------------------------
TR15727|c7_g3_i1_Potamotrygon_amandae       SAAQLPFS-----FVSVVVL-----QLVIK------------LVNFQ-S-----------
TR10580|c4_g1_i1_Potamotrygon_falkneri      SAAQLPFS-----FVSVVVL-----QLVIK------------LVNFQ-S-----------
XP_007896746_Callorhinchus_milii            SAPPLPLL----LF-TLVAF-----LLVVQ------------NLNF--------------
XP_006012182_Latimeria_chalumnae            GT--ADLI----MHLTLII---------L--------------IIVL------I---S-Q
XP_009685663_Struthio_camelus_australis     RKQKLYIF----LLGAVQLI-----LLYTA------------H-----------------
XP_010213407_Tinamus_guttatus               RKQKLHIF----LLGPTQLL-----LLLLH------------TIFFL-K-----------
XP_008943867_Merops_nubicus                 RKQKLYVF----LLGAMQLL-----LLHTT------------H-----------------
XP_013054655_Anser_cygnoides_domesticus     RKQKLYVF----LLGAIQLL-----LLYTA------------Y-----------------
NP_001295181_Gallus_gallus                  RKQKLYVI----LLGAMQLF-----LLYTA------------Y-----------------
XP_006024588_Alligator_sinensis             --QVANFF----LLGAMQIF-----LLFIA------------L-----------------
XP_006268718_Alligator_mississippiensis     --QVANFF----LLGAMQIF-----LLFIA------------L-----------------
XP_005305767_Chrysemys_picta_bellii         KNKMLNFL----LFGSMQVF-----LLLII------------H-----------------
EMP32607_Chelonia_mydas                     KNQMLNFL----LFGSMQVF-----LLLTV------------Q-----------------
XP_006122657_Pelodiscus_sinensis            KNQMLNIL----LFGSIQVF-----LFLIL------------H-----------------
XP_006633469_Lepisosteus_oculatus           NRIMKGML----FTPSLHIS-------CLS------------LIVFL-CLCLIV---K-C
XP_012678206_Clupea_harengus                DGFLGDLL----FVLSVHFS-------CLT------------IIVFL-GLCLLI---K-C
NP_001074140_Danio_rerio_                   DSILGELL----FVLSLHFS-------CLS------------VIMFL-GLCLLI---K-C
XP_007246617_Astyanax_mexicanus             DSFLPELL----LVLSLYFS-------CLS------------VIIFL-GLCLII---K-C
XP_005810535_Xiphophorus_maculatus          KSLVAHIM----LLLSVHFP-------CLC------------IIMFL-GLCLVI---K-G
XP_003449275_Oreochromis_niloticus          INLLGDIL----LLLSLHFF-------CLC------------VIIFL-GLCLII---K-S
XP_003967307_Takifugu_rubripes              TNLLADVL----IFLSVHFS-------CLC------------VIIFL-GLCLII---K-C
ACI32917_Salmo_salar                        --WKVWSV----LTLLLPLG-------LLT------------VLH---------------
NP_001119986_Xenopus_tropicalis             --GPCGIV----LVVSLVAL-------ILA------------LL----------------

EOB06026_Anas_platyrhynchos                 ------------------------------------------------------------
EMC77798_Columba_livia                      ------------------------------------------------------------
XP_002190553_Taeniopygia_guttata            EYNTVTAASSYDLEENDTRTFSS---SSSYKIRMFNLFHLILLLRALIQMIHESENILFP
BAM66299_Pterois_antennata                  MYVVMWK-----------------------------------------------------
BAM66300_Pterois_volitans                   MYVVMWK-----------------------------------------------------
AAO74499_Synanceia_horrida                  ------------------------------------------------------------
BAJ54082_Synanceia_verrucosa                MYIVM-------------------------------------------------------
NP_001094250_Rattus_norvegicus              ------------------------------------------------------------
XP_001370718_Monodelphis_domestica          ------------------------------------------------------------
XP_001502439_Equus_caballus                 ------------------------------------------------------------
XP_002752104_Callithrix_jacchus             ------------------------------------------------------------
XP_002818436_Pongo_abelii                   ------------------------------------------------------------
AAI04791_Homo_sapiens                       ------------------------------------------------------------
XP_527872_Pan_troglodytes                   ------------------------------------------------------------
XP_003217668_Anolis_carolinensis            MWATTEGP-GGEVPVFCPGTFRELHELAKKKRKKWKQMKM--------------------
ABI33944_Bitis_arietans                     ------------------------------------------------------------
ABI33937_Echis_ocellatus                    ------------------------------------------------------------
ABI33938_Cerastes_cerastes                  ------------------------------------------------------------
ABI33941_Echis_pyramidum_leakeyi            ------------------------------------------------------------
TR15727|c7_g3_i1_Potamotrygon_amandae       ------------------------------------------------------------
TR10580|c4_g1_i1_Potamotrygon_falkneri      ------------------------------------------------------------
XP_007896746_Callorhinchus_milii            ------------------------------------------------------------
XP_006012182_Latimeria_chalumnae            LIIRL-------------------------------------------------------
XP_009685663_Struthio_camelus_australis     ------------------------------------------------------------
XP_010213407_Tinamus_guttatus               ------------------------------------------------------------
XP_008943867_Merops_nubicus                 ------------------------------------------------------------
XP_013054655_Anser_cygnoides_domesticus     ------------------------------------------------------------
NP_001295181_Gallus_gallus                  ------------------------------------------------------------
XP_006024588_Alligator_sinensis             ------------------------------------------------------------
XP_006268718_Alligator_mississippiensis     ------------------------------------------------------------
XP_005305767_Chrysemys_picta_bellii         ------------------------------------------------------------
EMP32607_Chelonia_mydas                     ------------------------------------------------------------
XP_006122657_Pelodiscus_sinensis            ------------------------------------------------------------
XP_006633469_Lepisosteus_oculatus           LLFR--------------------------------------------------------
XP_012678206_Clupea_harengus                LIL---------------------------------------------------------
NP_001074140_Danio_rerio_                   LIL---------------------------------------------------------
XP_007246617_Astyanax_mexicanus             LIL---------------------------------------------------------
XP_005810535_Xiphophorus_maculatus          LILQTSKI-S--------------------------------------------------
XP_003449275_Oreochromis_niloticus          LIL---------------------------------------------------------
XP_003967307_Takifugu_rubripes              LTL---------------------------------------------------------
ACI32917_Salmo_salar                        ------------------------------------------------------------
NP_001119986_Xenopus_tropicalis             ------------------------------------------------------------
```

EOB06026_Anas_platyrhynchos	---
EMC77798_Columba_livia	---
XP_002190553_Taeniopygia_guttata	DYI
BAM66299_Pterois_antennata	---
BAM66300_Pterois_volitans	---
AAO74499_Synanceia_horrida	---
BAJ54082_Synanceia_verrucosa	---
NP_001094250_Rattus_norvegicus	---
XP_001370718_Monodelphis_domestica	---
XP_001502439_Equus_caballus	---
XP_002752104_Callithrix_jacchus	---
XP_002818436_Pongo_abelii	---
AAI04791_Homo_sapiens	---
XP_527872_Pan_troglodytes	---
XP_003217668_Anolis_carolinensis	-YI
ABI33944_Bitis_arietans	---
ABI33937_Echis_ocellatus	---
ABI33938_Cerastes_cerastes	---
ABI33941_Echis_pyramidum_leakeyi	---
TR15727|c7_g3_i1_Potamotrygon_amandae	---
TR10580|c4_g1_i1_Potamotrygon_falkneri	---
XP_007896746_Callorhinchus_milii	---
XP_006012182_Latimeria_chalumnae	---
XP_009685663_Struthio_camelus_australis	---
XP_010213407_Tinamus_guttatus	---
XP_008943867_Merops_nubicus	---
XP_013054655_Anser_cygnoides_domesticus	---
NP_001295181_Gallus_gallus	---
XP_006024588_Alligator_sinensis	---
XP_006268718_Alligator_mississippiensis	---
XP_005305767_Chrysemys_picta_bellii	---
EMP32607_Chelonia_mydas	---
XP_006122657_Pelodiscus_sinensis	---
XP_006633469_Lepisosteus_oculatus	---
XP_012678206_Clupea_harengus	---
NP_001074140_Danio_rerio_	---
XP_007246617_Astyanax_mexicanus	---
XP_005810535_Xiphophorus_maculatus	---
XP_003449275_Oreochromis_niloticus	---
XP_003967307_Takifugu_rubripes	---
ACI32917_Salmo_salar	---
NP_001119986_Xenopus_tropicalis	---

Figure S14: Summary of the *P. amandae* and *P. falkneri de novo* whole transcriptome analysis workflow.

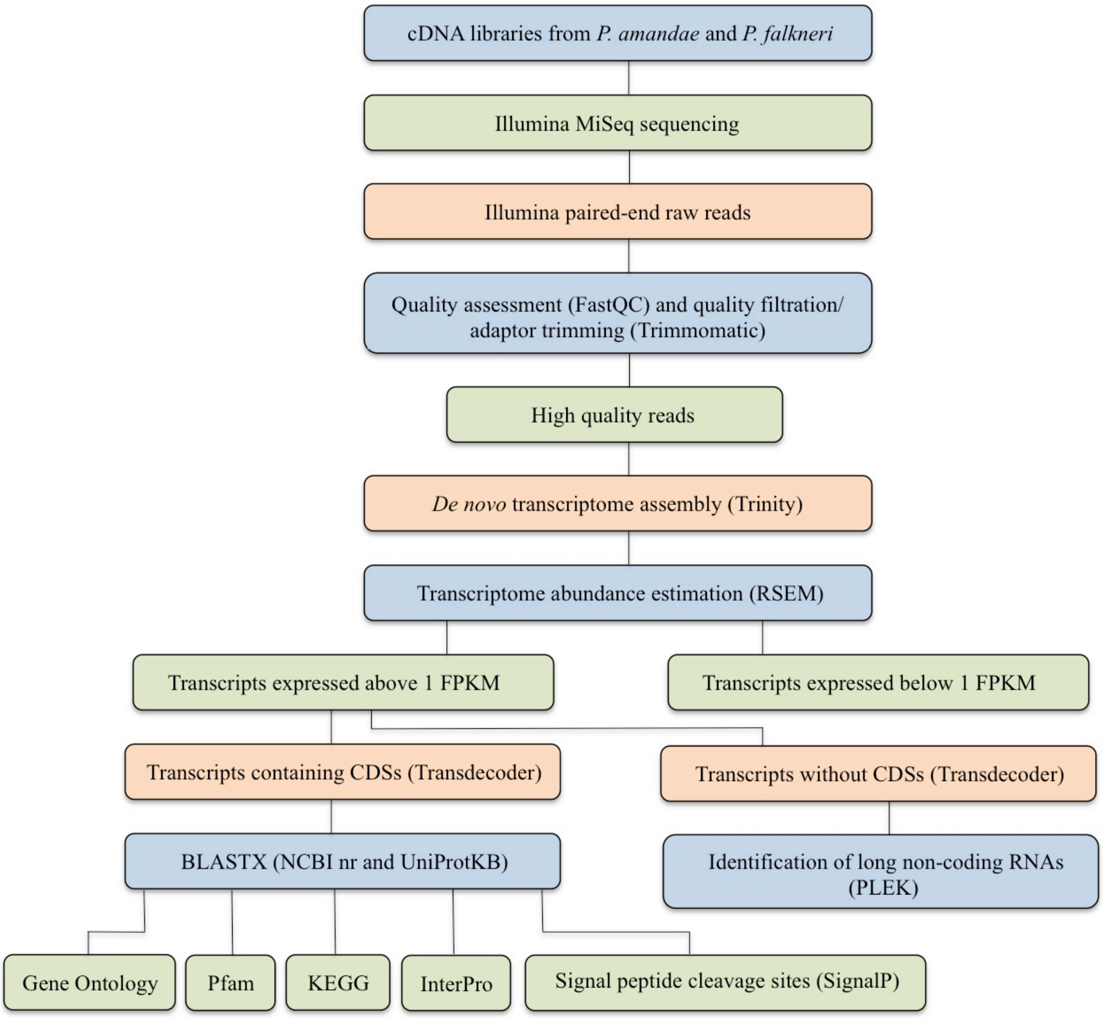

Supplementary tables:

Table S1. The 25 most abundant InterPro entries encountered in the *P. amandae* assembled transcriptome.

InterPro Accession	InterPro Description	Counts
IPR027417	P-loop containing nucleoside triphosphate hydrolase	1168
IPR011009	Protein kinase-like domain	746
IPR015880	Zinc finger, C2H2-like	710
IPR013083	Zinc finger, RING/FYVE/PHD-type	690
IPR000719	Protein kinase domain	661
IPR007087	Zinc finger, C2H2	642
IPR016024	Armadillo-type fold	587
IPR013783	Immunoglobulin-like fold	549
IPR011993	Pleckstrin homology-like domain	540
IPR015943	WD40/YVTN repeat-like-containing domain	526
IPR013087	Zinc finger C2H2-type/integrase DNA-binding domain	508
IPR017986	WD40-repeat-containing domain	479
IPR001841	Zinc finger, RING-type	477
IPR012677	Nucleotide-binding alpha-beta plait domain	422
IPR001849	Pleckstrin homology domain	419
IPR000504	RNA recognition motif domain	390
IPR002290	Serine/threonine/dual specificity protein kinase, catalytic domain	370
IPR013320	Concanavalin A-like lectin/glucanase domain	327
IPR011989	Armadillo-like helical	324
IPR007110	Immunoglobulin-like domain	320
IPR001452	SH3 domain	306
IPR011992	EF-hand domain pair	263
IPR029071	Ubiquitin-related domain	253
IPR000008	C2 domain	229
IPR011011	Zinc finger, FYVE/PHD-type	228

Table S2. The 25 most abundant InterPro entries encountered in the *P. falkneri* assembled transcriptome.

InterPro Accession	InterPro Description	Counts
IPR027417	P-loop containing nucleoside triphosphate hydrolase	1076
IPR013083	Zinc finger, RING/FYVE/PHD-type	679
IPR011009	Protein kinase-like domain	674
IPR000719	Protein kinase domain	602
IPR016024	Armadillo-type fold	570
IPR015880	Zinc finger, C2H2-like	549
IPR011993	Pleckstrin homology-like domain	519
IPR007087	Zinc finger, C2H2	495
IPR001841	Zinc finger, RING-type	475
IPR015943	WD40/YVTN repeat-like-containing domain	454
IPR013783	Immunoglobulin-like fold	452
IPR017986	WD40-repeat-containing domain	417
IPR001849	Pleckstrin homology domain	396
IPR013087	Zinc finger C2H2-type/integrase DNA-binding domain	379
IPR012677	Nucleotide-binding alpha-beta plait domain	364
IPR000504	RNA recognition motif domain	329
IPR002290	Serine/threonine/dual specificity protein kinase, catalytic domain	325
IPR011989	Armadillo-like helical	319
IPR001452	SH3 domain	305
IPR013320	Concanavalin A-like lectin/glucanase domain	281
IPR007110	Immunoglobulin-like domain	265
IPR011992	EF-hand domain pair	252
IPR029071	Ubiquitin-related domain	243
IPR011011	Zinc finger, FYVE/PHD-type	235
IPR000008	C2 domain	216

Table S3: Theoretical physicochemical parameters obtained for the modeled proteins.

Models	PI	Disulfide bonds	SASA (Å^2)	Helix (%)	b-sheet (%)	Loop (%)
**Phospholipase A2**						
P. falkneri	9.19	7	8025.41	38.4	8.8	52.8
P. amandae	8.72	7	7766.59	38.4	8.8	52.8
**Hyaluronidase**						
P. falkneri	7.76	2	15860.55	38.6	15.4	46.0
P. amandae	7.76	2	15906.31	38.6	15.4	46.0
**CRISP**						
P. falkneri	7.82	0	9001.23	31.5	17.8	50.7
P. amandae	9.1	0	12119.23	22.4	10.9	66.7
**L-amino acid oxidase**						
P. falkneri	8.53	0	27563.87	28.6	16.7	54.7
P. amandae	8.43	0	28589.10	31.0	21.0	46.0
**Serine protease**						
P. falkneri	9.54	4	13587.19	4.7	39.5	55.8
P. amandae	5.54	3	12570.19	1.6	44.4	54.0
**VEGFA**						
P. falkneri	7.31	3	9219.11	7.5	38.3	54.2
**Metalloproteinase**						
P. amandae	5.81	1	9744.49	36	21	41

PI: Theoretical isoelectric point; **SASA**: solvent-accessible surface area; **Å**: Angstrom.

Table S4: UniprotKB acession numbers of the protein sequences selected for multiple sequence alignment

UniProtKB accession number	Species
XP_013054655	*Anser cygnoides domesticus*
XP_008943867	*Merops nubicus*
XP_005305767	*Chrysemys picta bellii*
XP_009685663	*Struthio camelus australis*
XP_010213407	*Tinamus guttatus*
XP_012678206	*Clupea harengus*
XP_007896746	*Callorhinchus milii*
AAI04791	*Homo sapiens*
AAO74499	*Synanceia horrida*
ABI33937	*Echis ocellatus*
ABI33938	*Cerastes cerastes*
ABI33941	*Echis pyramidum leakeyi*
ABI33944	*Bitis arietans*
ACI32917	*Salmo salar*
BAJ54082	*Synanceia verrucosa*
BAM66299	*Pterois antennata*
BAM66300	*Pterois volitans*
EMC77798	*Columba livia*
EOB06026	*Anas platyrhynchos*
NP_001074140	*Danio rerio*
NP_001094250	*Rattus norvegicus*
NP_001119986	*Xenopus tropicalis*
NP_001295181	*Gallus gallus*
XP_001370718	*Monodelphis domestica*
XP_001502439	*Equus caballus*
XP_002190553	*Taeniopygia guttata*
XP_002752104	*Callithrix jacchus*
XP_002818436	*Pongo abelii*
XP_003217668	*Anolis carolinensis*
XP_003449275	*Oreochromis niloticus*
XP_003967307	*Takifugu rubripes*
XP_005810535	*Xiphophorus maculatus*
XP_006012182	*Latimeria chalumnae*
XP_006024588	*Alligator sinensis*
XP_006122657	*Pelodiscus sinensis*
XP_006268718	*Alligator mississippiensis*
XP_006633469	*Lepisosteus oculatus*
EMP32607	*Chelonia mydas*
XP_007246617	*Astyanax mexicanus*
XP_527872	*Pan troglodytes*
